# Spinal cord injury: molecular mechanisms and therapeutic interventions

**DOI:** 10.1038/s41392-023-01477-6

**Published:** 2023-06-26

**Authors:** Xiao Hu, Wei Xu, Yilong Ren, Zhaojie Wang, Xiaolie He, Runzhi Huang, Bei Ma, Jingwei Zhao, Rongrong Zhu, Liming Cheng

**Affiliations:** 1 Division of Spine, Department of Orthopaedics, Tongji Hospital, Tongji University School of Medicine, 200065 Shanghai, China; 2 Key Laboratory of Spine and Spinal cord Injury Repair and Regeneration (Tongji University), Ministry of Education, 200065 Shanghai, China; 3 Clinical Center For Brain And Spinal Cord Research, Tongji University, 200065 Shanghai, China

**Keywords:** Regeneration and repair in the nervous system, Trauma

## Abstract

Spinal cord injury (SCI) remains a severe condition with an extremely high disability rate. The challenges of SCI repair include its complex pathological mechanisms and the difficulties of neural regeneration in the central nervous system. In the past few decades, researchers have attempted to completely elucidate the pathological mechanism of SCI and identify effective strategies to promote axon regeneration and neural circuit remodeling, but the results have not been ideal. Recently, new pathological mechanisms of SCI, especially the interactions between immune and neural cell responses, have been revealed by single-cell sequencing and spatial transcriptome analysis. With the development of bioactive materials and stem cells, more attention has been focused on forming intermediate neural networks to promote neural regeneration and neural circuit reconstruction than on promoting axonal regeneration in the corticospinal tract. Furthermore, technologies to control physical parameters such as electricity, magnetism and ultrasound have been constantly innovated and applied in neural cell fate regulation. Among these advanced novel strategies and technologies, stem cell therapy, biomaterial transplantation, and electromagnetic stimulation have entered into the stage of clinical trials, and some of them have already been applied in clinical treatment. In this review, we outline the overall epidemiology and pathophysiology of SCI, expound on the latest research progress related to neural regeneration and circuit reconstruction in detail, and propose future directions for SCI repair and clinical applications.

## Introduction

SCI is defined as damage to the spinal cord that causes temporary or permanent changes in its function, and this condition has a high incidence, high costs, a high disability rate and a low age of onset.^1^ SCI can be caused by high-intensity injuries, such as traffic accidents, falling injuries and violent injuries, or by infections, tumors, vertebral column degenerative disorders, ischemia–reperfusion injuries, and vascular causes.^2,3^ Serious SCI represents a significant physical, psychological and financial burden for patients and their families. It was reported that there are 759,302 patients with traumatic SCI in total and 66,374 new cases annually in China.^4^ In addition, data from the United States showed that the annual incidence of SCI is approximately 17,000 people per year, and the first-year expenses of one high tetraplegia patient are more than $1 million.^1^

According to the cause of injury, SCI can be divided into traumatic and nontraumatic SCI. According to the pathophysiology, acute SCI can be divided into primary and secondary injuries.^5^ According to the severity, SCI can be divided into complete or incomplete injury, and incomplete SCI can manifest as central core syndrome, Brown-Séquard syndrome, anterior cord syndrome, and posterior core syndrome.^5^ SCI has always been a research hotspot in the field of neural regeneration and repair. We selected the Web of Science (core collection) as the data source, and 35,567 documents related to SCI research were retrieved from January 1, 2012, to January 1, 2022. The retrieval strategy was: ((TS=spinal cord injury) OR (TS= spinal cord injuries) OR (TS= spinal cord traum*) OR (TS=spinal traum*)). From January 1, 2012 to January 1, 2022, after deleting repetition, 35,567 documents were retrieved. The document type mainly included “Article” and “Review”. Then the retrieval results were input into the bibliometric analysis software to further analyze. The Bibiliometrix R package provided a suite of tools for bibliometric studies, which was selected as the analysis software in bibliometric analysis.^6^ It was an open-source statistical programming environment, based on R language with a large number of efficient high-quality statistical algorithms and integrated visualization tools. Among these published articles, pathological mechanism studies and comprehensive studies, including surgical, cell transplantation and materials construction studies, and clinical trials for potential clinical translation were the most common.

**Fig. 1:**
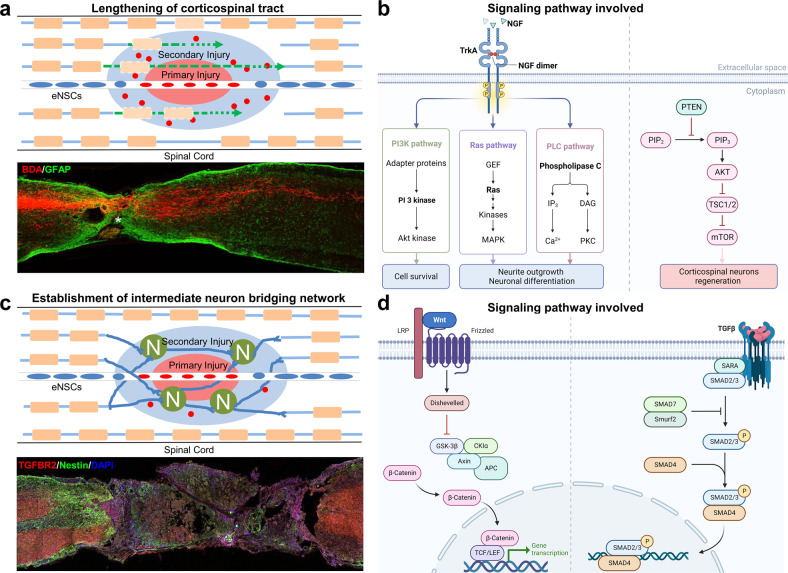
Two main repair strategies and related signaling pathway of neural circuit reconstruction after SCI. **a** Type I: CST extension. PTEN and suppressor of cytokine signaling 3 (SOCS3) deletion could effectively enhance the CST traced with biotin dextran amine (BDA), which showed the regenerative ability of adult corticospinal neurons.^13,513^ **b** Signaling pathways involved such as PI3K pathway, Ras pathway, PLC pathway, and PTEN/mTOR pathway. The figure was created with BioRender.com. **c** Type II: establishment of intermediate neuron bridging network. SCI mice were significantly improved by LDH-NT3 implantation.^34^ **d** Signaling pathways involved such as Wnt/β-Catenin pathway and TGFβ/SMAD pathway. The figure was created with BioRender.com

The recovery of spinal cord function depends on the remodeling and integrity of neural circuits. After SCI, the breakage of neuronal axons and the death of neurons cause dysfunction of neural circuits. The plasticity of neural circuits is the basis of the recovery of neural function. The traditional repair principle is to promote the regeneration and extension of the corticospinal tract (CST) and re-establish the connection with distal neurons, including reducing the production of regenerative-related inhibitors, such as chondroitin sulfate proteoglycans (CSPG)/NogoA/myelin-associated glycoprotein (MAP)/oligodendrocyte myelin glycoprotein (OMG), and even lipid metabolites, in the microenvironment during the early stage of SCI or promoting axon regeneration by exploiting the intrinsic growth ability.^7–10^ Phosphatase and tensin homolog (PTEN) deletion effectively enhanced the regenerative ability of adult corticospinal neurons and promoted the recovery of motor function after SCI.^11–13^ However, regenerated axons can hardly be reconnected to distal effectors because of the long distance. Recently, interneurons, which may provide bridges to proximal and distal neurons and form new neural circuits, have become an important strategy for SCI repair. These crucial interneurons may be derived from primitive interneurons in the spinal cord or cells differentiated from transplanted stem cells and endogenous NSCs (eNSCs) (Fig. 1).^14–23^

The remodeling of neural circuit is extremely difficult, and the analysis of pathological mechanism of SCI helps to identify more key intervention targets. The primary injury of SCI is unpredictable and irreversible, while the secondary injury is a target for therapeutic intervention and should be regarded as an important regulatory period for the treatment of SCI.^24–27^ However, the pathological mechanisms of SCI are like a “black box” and are still not entirely clear. Furthermore, the role of each pathological mechanism in SCI, such as the immune response and astrocyte scar formation, is controversial. Recently, transcriptome analyses, weighted gene coexpression network analysis (WGCNA) and single-cell sequencing technology have been widely used in SCI research and have provided better tools for clarifying pathological mechanisms.^18,28–31^ By using single-cell RNA sequencing, Frisén found that neural stem cells (NSCs) in the ependymal region of the spinal cord had latent lineage potential to differentiate into oligodendrocytes, which could be realized by regulating the expression of OLIG2 in NSCs.^32^ Our previous work revealed through transcriptomic analyses and WGCNA that a steady state of immune deficiency potentially led to CNS hyperconnectivity and then revealed using single-cell sequencing that temporal and spatial cellular and molecular pathological alterations occurred in the adult spinal cord after injury.^28,33^ Some researchers established a detailed transcriptomic profile of every major cell type in a mouse SCI model at the single-cell level and revealed novel insights into myeloid cell heterogeneity and specific signaling pathways by which unique myeloid subtypes contribute to the wound-healing process in the CNS.^31^

Whether transplanted stem cells or eNSCs, in the process of neural circuit remodeling after SCI, they are similar to “seeds”, requiring good regeneration microenvironment and necessary means of cell fate regulation, such as “soil” and “fertilizer”. To date, there have been many studies on the regulation of the injury microenvironment by bioactive materials, which combined with single or multiple neurotrophic factors to not only reduce the local immune response but also mobilize eNSCs to repair SCI.^23,34,35^ In terms of cell fate regulation, novel biomolecules and physical regulation have received more attention and have become noninvasive means for SCI repair.^36–38^ Indeed, most bioactive materials and physical regulation methods aim to repair SCI by promoting the plasticity of intermediate neural network.^23,34,39^

This review will summarize the latest research progress on SCI, including epidemiology and pathophysiology, the current research progress on neural regeneration and SCI repair, the progress of clinical trials and the prospects of clinical translation research on SCI.

## Overall incidence and demographic characteristics

Since the 1980s, the incidence of SCI in different countries or regions has been reported.^40^ When we summarized the incidence of SCI for the five continents, we found that there was no significant difference in the incidence of SCI between continents. Comparing the studies from the Americas and Asia with the largest sample sizes, the incidence of SCI was 54 cases per million people in the United States,^1^ as a representative country in the Americas, and 66.4 cases per million people in China,^4^ as a representative county in Asia. Germany^41^ and Australia^42^ in Europe and Oceania, respectively, had 74.8 and 32.3 cases per million people. In the few African^43–45^ countries that reported the incidence of SCI, there were no significant differences in the rates of SCI in these countries. Regarding the etiology and demographic characteristics of SCI, motor vehicle accidents (MVAs) and falls are the most common causes of injury, while an age younger than 50 years and male sex are demographic risk factors for SCI.^1,46–54^ Table 1 shows a summary of epidemiologic investigations and demographics of SCI patients. Overall, a wide range of SCI incidence rates among different countries has been observed, and some developing countries show a low SCI incidence rate, while some developed countries show a high SCI incidence rate.^46,55,56^

**Table 1:** The synthetic view of epidemiologic investigation and demographics of SCI patients

Location	Research period	Incidence	Prevalence	Leading causes	Mean age	Sex ratio
Australia^42^	1921–2011	32.3	490	N/A	N/A	4.00:1
Austria^481^	2002–2012	17	N/A	Falls	N/A	1.86:1
Botswana^384^	2011–2013	13	N/A	MVCs	N/A	2.45:1
Brazil^482^	1997–2006	26.1	N/A	Falls	36.75	7.35:1
Cambodia^483^	2013–2014	N/A	N/A	MVCs	37	5.20:1
Canada^484^	2000–2011	16.9	N/A	MVCs	46.2	3.95:1
China^4^	2010–2013	66.4	759.3	Falls	43.7	1.01:1
Egypt^485^	2009–2012	N/A	180	MVCs	40	5.00:1
Estonia^486^	1997–2007	39.7	N/A	Falls	39	5.45:1
Ethiopia^45^	2008–2012	N/A	N/A	MVCs	31.7	7.59:1
Europe^487^	1988–2009	N/A	N/A	MVCs	44.5	1.85:1
Finland^488^	2007–2011	27	N/A	Falls	N/A	N/A
Germany^41^	2002–2012	74.8	N/A	MVCs	48.9	N/A
Ghana^44^	2012–2014	N/A	N/A	MVCs	36.3	3.2:1
Iceland^489^	1975–2009	33.5	526	MVCs	38	2.57:1
India^490^	2000–2008	N/A	N/A	Falls	N/A	4.20:1
Iran^491^	2003–2008	44‘	440	MVCs	31	1.00:1
Ireland^492^	2000	13.1	N/A	MVCs	37	6.69:1
Italy^493^	2011–2022	26.5	N/A	MVCs	59.2	2.15:1
Japan^494^	2011–2012	121.4	N/A	Falls	67.6, 64.3	2.65, 2.75:1
Korea^495^	2004–2008	N/A	N/A	MVCs	43.6	2.86:1
Kuwait^496^	2010–2015	N/A	N/A	MVCs	36.4	4.3:1
Macedonia^497^	2015–2016	13	180	MVCs	43	5.3:1
Malaysia^498^	2006–2009	N/A	N/A	MVCs	39	3.35:1
Nepal^499^	2008–2011	N/A	N/A	Falls	N/A	2.77:1
Netherlands^49^	2010	14	N/A	Falls	62	2.85:1
Nigeria^500^	2009–2012	N/A	N/A	MVCs	36.1	4.31:1
Norway^501^	2009–2012	16.5	N/A	N/A	51	2.2:1
Pakistan^502^	2008–2012	10.23	N/A	MVCs	20–29	4.25:1
Russia^503^	2012–2016	16.6	N/A	MVCs	42.1	2.4:1
Saudi Arabia^504^	2003–2008	N/A	N/A	MVCs	29.5	7.53:1
South Africa^43^	2013–2014	20	N/A	MVCs	48.0	3.33:1
Spain^505^	2001–2015	9.3	N/A	Falls	42.8	4.03:1
Switzerland^506^	2005–2012	18	N/A	Falls	48	2.90:1
Tanzania^507^	2010–2012	26	N/A	Falls	39.1	4.19:1
Turkey^52^	2013–2014	21.3	N/A	Falls	38.3	2.31:1
USA^1^	2015–2022	54	299	MCVs	43	3.55:1

Note. Only the most representative study with the largest sample size in each region is retained in the table. Incidence rate represents the frequency of new cases of TSCI occurring in a given population within a given period of time, while the prevalence rate is the proportion of a population found to have the TSCI condition

## Pathophysiological mechanism of SCI

The pathophysiology of SCI includes primary injury and secondary injury; the former is usually a mechanical injury to the cord, and the latter is the consequence of cell and biological reactions to the primary injury, which involve the immune system, nervous system, vascular system, etc., including hemorrhage, ischemia, oxidative stress, inflammatory reaction, neural cell death, demyelination, scar formation and so on. At present, there are many animal models mimics human SCI, including zebrafish, rodents, large animals and primates. They have their own advantages and disadvantages in the process of studying SCI. Among them, zebrafish has strong nerve regeneration ability and is suitable for studying transverse injury models. Studies have shown that the mechanism of nerve regeneration in the zebrafish SCI model may be related to extracellular matrix Cthrc1 or pro-regenerative macrophages.^57,58^ Rodents, such as mice and rats, are most widely used because of its good repeatability, and are suitable for contusion or crush or suction models.^59^ Large animals and primates are generally suitable for the study of spinal cord hemisection model due to nursing difficulties.^60^ In a study on the pathological mechanism of the model of spinal cord hemisection injury in nonhuman primates, it reported that activated microglia/macrophages were found both within the injury center and the peri lesion area, and in contrast to rodent, substantial reactive astrocytic responsesat the lesion border were not observed in the monkey. Conversely, a deposit of robust fibrotic scar was observed at the injury epicenter, which filled the space originally created by the hemisection.^61^

**Fig. 2:**
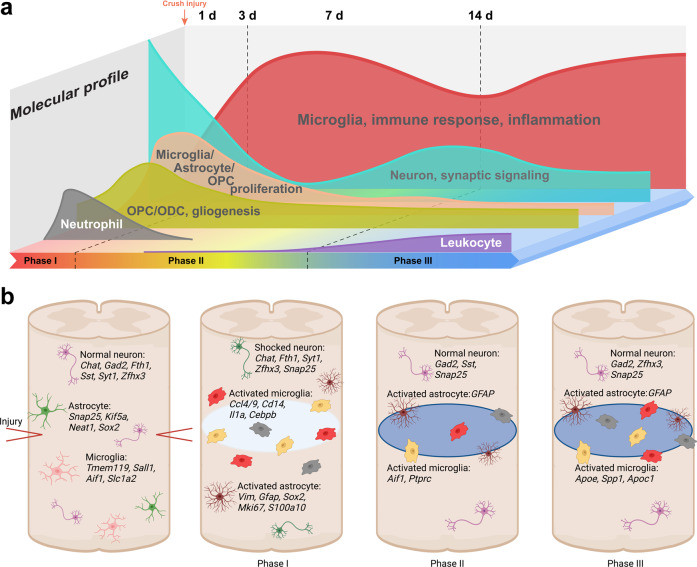
A schematic illustration of molecular (**a**) and cellular (**b**) changes post SCI.^33^

Our previous studies reported temporal molecular and cellular changes in crush-injured adult mouse spinal cord using single-cell transcriptomic analyses combined with classic anatomical, behavioral, electrophysiological analyses.^33^ We found that most dynamic changes occur at 3 days post injury, and by day-14 the second wave of microglial activation emerged, accompanied with changes in various cell types including neurons, indicative of the second round of attacks. By day-38, major cell types are still substantially deviated from uninjured states, demonstrating prolonged alterations.^33^ It was reported that the spinal ischemia, vasogenic edema and glutamate excitotoxicity were mean involved in the acute stage of SCI, while neuroinflammatory, mitochondrial phosphorylation, production of NOS in the subacute stage, and apoptosis and necrosis, axon degeneration, axon remyelination, axon remodeling, glial scar formation in the chronic stage (Fig. 2).^62^

The cell response is the basic unit in the pathophysiology of SCI and has temporal and spatial characteristics.^33,63^ The elucidation of the cellular response, especially the response mechanisms of different cell subsets, is of great significance for finding effective intervention targets for SCI. The pathological mechanism of SCI discussed in this review mainly focuses on rodents.

### Immune responses at the peri-injury site

The immune response is a critical pathological mechanism that may determine the prognosis of SCI. When SCI occurs, local microglial reactivity and the destruction of the blood‒spinal cord barrier provide opportunities for blood-derived immune cells, including neutrophils, monocyte macrophages, and lymphocytes, to enter spinal cord tissue. These cells secrete proinflammatory or immunomodulatory factors to participate in the immune response. The immune response has been thought to have destructive effects and is not conducive to SCI repair. However, the roles of the immune response in SCI damage expansion and regeneration and repair are still controversial.

#### In situ immune cells

##### Microglia

When SCI occurs, microglia change their cell morphology and protein expression profile, making it easier to phagocytize and remove debris, and they change to a proinflammatory state overall and mediate further secondary injury.^64^ When microglia are eliminated long-term in the injured spinal cord, the transcriptional inflammatory response is reduced.^65^ The microglial response began in the early stage after SCI and lasted for up to 1 year in a rat transection SCI model.^66^ There may be two peaks of microglial activation. Nguyen et al. reported that in a contusion model of SCI in rats, activated microglia were detected with two peak times, one at 7 dpi and one at 60 dpi.^67^ In our previous studies, we found similar characteristics of microglia, with two peaks in a mouse SCI model.^33^ As in spatial distribution, in a pathological study of human SCI, it was reported that microglia rapidly disappeared in the lesion core. In contrast, at the peripheral margin, the number of TMEM119^+^ microglia is maintained through local proliferation and exhibits a major proinflammatory phenotype.^68^ These results were consistent with that reported in other literature.^69^

The function of microglia depends on their phenotype, which changes in response to the microenvironment. Microglia have two main phenotypes: the M1 phenotype, which tends to promote the inflammatory response and aggravate neuroinflammation, and the M2 phenotype, which tends to exert anti-inflammatory effects and promote tissue repair. It was reported that transplantation of M2-polarized microglia could promote recovery of motor function in a mouse SCI model.^70^ However, more subtypes of microglia may exist in the injured spinal cord. In our previous studies, we found a total of 8 clusters of microglial subpopulations in SCI mice at single-cell resolution, and each subpopulation had different characteristics; this topic needs further research.^33^ The best time window for the conversion of reactive microglia to their neuroprotective phenotypes may be the first week post SCI.^71^ The phenotypic transformation of microglia is dynamic and regulated by many factors in the injured microenvironment. Recently, researchers showed that LRCH1 alleviates the activation of p38 MAPK and Erk1/2 signaling and negatively regulates microglia-mediated neuroinflammation after SCI.^72^ Downregulation of ubiquitin-specific protease 4 expression promotes microglial activation through NF-κB by attenuating the deubiquitination of TRAF6.^73^

Interestingly, more and more studies have found that microglia may become the key target cells for the repair of SCI. Microglia are involved in the formation of corralling and glial scarring, which reduce parenchymal immune infiltrates and reduce the apoptosis of neurons and oligodendrocytes in the first two weeks after SCI.^74–76^ It has also been reported that in a neonatal mouse SCI model, the spinal cord can heal without scarring and allow long projection axons to grow through the lesion, while the depletion of microglia destroys this healing and prevents axon regeneration.^77^ The authors point out that microglia temporarily secrete fibronectin and its binding protein to form an extracellular matrix bridge connecting the broken ends of axons. In addition, the unique role of neonatal microglia is related to the expression of peptidase inhibitors. Both adult microglia and neonatal microglia treated with peptidase inhibitors can significantly improve healing and axon regeneration after transplantation in the setting of adult SCI.^77^ Our previous study also found that there are microglial subsets in the adult spinal cord, which are similar to those in neonatal mice, but their ability to promote regeneration is reduced due to the expression of higher levels of CD68 and lower levels of p2ry12.^33^

#### Blood-derived immune cells

##### Neutrophils

Neutrophils are considered to be one triggering factor of the secondary injury process after SCI. Their recruitment are facilitated by interleukin 1a (IL-1a), IL-β, IL-8, tumor necrosis factor (TNF), granulocyte colony-stimulating factor, CCL3, CXCL1, CXCL2, and CXCL5, which are secreted by resident cells of the spinal cord, after detecting the damage. Meanwhile, the neutrophils could recognize these pathogen-associated molecular patterns (PAMPs) or danger-associated molecular patterns (DAMPs) through pattern recognition receptors (PRRs) and its downstream signaling pathways such as nuclear factor kB (NF-kB) pathways^78^ Neutrophils can be detected in the spinal cord 2 h after injury and peak at 1 dpi in rats or 3 dpi in mice.^25,67^

Neutrophils generally participate in the pathological mechanism of SCI in a harmful role. On the one hand, they release a variety of proinflammatory mediators, including reactive oxygen species (ROS), lysosomal enzymes, proteolytic enzymes (such as elastase and matrix metalloproteinase-9) and oxidative enzymes (myeloperoxidase; MPO).^79–81^ It was reported that spleen tyrosine kinase could facilitate neutrophil activation and worsen long-term neurologic deficits after SCI.^82^ On the other hand, neutrophils can produce neutrophil extracellular traps (NETs), which aggravate secondary injury by promoting neuroinflammation and blood-spinal cord barrier destruction in SCI.^80,83–85^ However, Neutrophils can also be divided into subsets. Recently, research revealed a new subset of neutrophils, CD14^+^ly6glow granulocytes, that can promote spinal cord repair, partly due to secretion of the growth factors NGF and IGF-143.^82,86^

Recent studies have shown that inhibiting leukocyte infiltration contributes to functional recovery after SCI, and a high neutrophil-to-lymphocyte ratio is associated with poor outcomes in patients with acute cervical SCI.^87–89^ However, the early infiltration of neutrophils not only accurately guides circulating macrophages by secreting enzymes and other factors but also creates conditions that promote macrophage phagocytosis. In this regard, the role of neutrophils also has a favorable side.^78,90^

##### Myeloid monocytes

Monocyte-derived macrophages (MDMs) also have two main subgroups, M1 and M2 macrophages. In a contusion SCI model, M1 macrophages were detected at an early stage and maintained at a high level, while M2 macrophages were briefly detected high levels and returned to preinjury levels after 1 week.^91^ In related studies on the mechanism of macrophage activation after SCI, an increase in intracellular iron and myelin debris could regulate the polarization of macrophages, promoting a harmful M1 phenotype.^92,93^ The lipid catabolic pathway is another target for regulating the important functions of macrophages, which was revealed by detecting the specific transcription profile of macrophages after SCI.^30^

To distinguish the role of microglia from that of MDMs, the lysozyme M EGFP-knockin mouse provides a better tool for macrophage research in combination with some specific markers, such as P2ry12, Siglec H, TGFBR1, and Tmem119.^94–98^ As in time distribution, it was reported that microglia were the main type of macrophages during the early response to SCI, and infiltrating macrophages later became the main cells in contact with degenerative axons, which lasted for 42 days.^95^ As in spatial distribution, the MDMs were distributed at the center of the site of injury, while microglia were distributed at the edge of the lesion.^93^ This had also been confirmed in pathological studies of human SCI, which reported that in the lesion core, microglia were rapidly lost while intermediate (co-expressing pro- as well as anti-inflammatory molecules) blood-borne macrophages dominated.^68^ Interestingly, the distribution characteristics of different subsets of macrophages are also different. It was reported that Cx3Cr1^hi^ macrophages are present in the glial scar, whereas Cx3Cr1^lo^ macrophages are present in fibrotic scar, and this may drived distinct physiological processes.^69^

However, MDMs also participate in wound-healing activities after infiltrating the injury site. For example, they promote corralling and recovery via Plexin-B2, forming a closed loop surrounded by astrocytes, and Plexin-B2 ablation in myeloid cells impairs motor sensory recovery.^74^ Also, MDMs could provide a regulatory mechanism by inhibiting microglia-mediated phagocytosis and inflammation.^99^

After SCI, a large number of myelin sheath fragments and necrotic tissue are produced locally, which need to be cleared by phagocytes. Compared with activated microglial cells, blood-derived macrophages have stronger phagocytic capacity. However, after phagocytosis of a large amount of lipid-rich myelin sheath tissue, macrophages will form foam macrophages, which will not only reduce the phagocytosis ability, but also cause damage to neural tissue.^100^ It was reported that endogenous glucocorticoid receptors (GRs) signaling was a key pathway that normally inhibits mechanisms of macrophage-mediated repair after SCI through regulation of lipid and myelin phagocytosis and foamy macrophage formation.^101^

##### Lymphocytes

T lymphocytes and B lymphocytes are the main cell types involved in adaptive immunity. In human SCI, lymphocyte numbers were low and mainly consisted of CD8^+^ T cells.^68^ In the animal SCI model, T cells can be detected at 1 dpi after SCI and peak at 7 dpi, and sustained T-cell responses are observed at 180 dpi.^67^ Cytotoxic CD8^+^CD28^+^ T cells were dominant in the first two weeks, which means those two weeks after SCI, survival time can be prolonged and the proportion of CD8^+^ regulatory T cells can be increased.^102^ It was also reported that γδ T cells, a subgroup of T cells, provided an early source of IFN-γ, which aggravated the inflammatory response after SCI.^103,104^

Adaptive immunity is unfavorable to nerve recovery because T-cell- and B-cell-immunodeficient SCI models showed better neurological recovery.^28,105–107^ It has been reported that perforin derived from CD8 T cells damages the CNS by increasing the permeability of the blood-spinal cord barrier, resulting in the infiltration of inflammatory cells and related cytokines.^108^ In addition, T-cell infiltration and signal transduction in the dorsal spinal cord of adults are the main causes of neuropathic pain, such as hypersensitivity.^109^ However, myelin basic protein-activated T cells play a beneficial role in the repair of the CNS, and an increase in the number of Th2 cells promotes the transformation of Th1 and M1 cells to Th2 and M2 cells, respectively, thus changing the local microenvironment and facilitating the repair of SCI.^110^

### Neural cell-related responses and interactions

Astrocytes undergo long-term and large-scale activation and proliferation after SCI occurs. Due to the proliferation of astrocytes and the infiltration of inflammatory cells, neurons, oligodendrocytes and NSCs/ neural progenitor cells (NPCs) are vulnerable to degeneration and death. During the pathophysiological process of SCI, all neural functional cells produce pathological responses, and there is also abnormally active neural communication between neural cells and inflammatory cells. Analyses of the responses and communication mechanisms of these important functional cells are helpful for understanding the pathological mechanism of SCI and providing key regulatory targets.

#### Neurons and NSCs/NPCs

The loss of mature neurons is the main cause of functional defects after SCI, and this is directly involved in the induction of programmed cell death (PCD) in neural cells, including apoptosis, necroptosis, autophagy, and ferroptosis.^111^ It was reported that apoptosis in neurons could be detected at 4 h and peaked at 8 h after SCI, the total number of axons at the injured site decreased immediately, and the downward trend continued during the subacute phase and reached a minimum during the chronic phase.^112,113^ The results showed that cytoplasmic Nissl material was decreased in neurons within a few minutes after mild SCI, and the lesion area expanded and cavitated within the next 7 days.^112^ Oxidative stress is an important cause for ongoing neuronal damage long after initial trauma. In one study of human SCI pathology, oxidative neuronal cell body and axonal damage can be observed through the intracellular accumulation of amyloid precursor protein (APP) and oxidized phospholipid (e06), which occurs early in the lesion core and decreases over time. In contrast, within the peripheral margin, significant neuronal APP^+^/e06^+^ axonal dendritic damage was detected, which remained significantly elevated for months/years.^68^

Neuronal apoptosis has a close relationship with cell autophagy. Autophagy disorder causes the accumulation of neurotoxic proteins and subsequent neuronal cell death.^114^ Neuronal apoptosis is also closely related to regulatory proteins, such as the RNA-binding protein src-associated in mitosis (Sam68), IGFBP6, a member of the insulin-like growth factor-binding protein family, HS1-associated protein X-1 (HAX1) and TNF receptor-associated factor 7 (TRAF7).^115–118^ Ferroptosis is a novel type of iron-dependent cell death, and it has a strong correlation with secondary injury after SCI.^119^ The inhibition of ferroptosis could promote the recovery of neurological function by enhancing neuronal survival.^120^ Ferroptosis is different from other forms of cell death, and it may be a novel direction for further research on acute CNS injuries.^121^

After primary SCI injury, the rostral end of the axon retracts, and the caudal end loses the support of the cell body, resulting in degeneration and disintegration. However, at the same time, the axon starts regeneration and repair, but the molecular mechanism is unclear. It was reported that axonal sprouting was observed within 6 h after SCI, which may be supported by calpain activation and protein synthesis in axons.^122^ The author further reported that the accumulation of damaged axon glial complexes (AGCs) was an obstacle for axon regeneration at the injury site. When the author surgically eliminated the AGC, the regenerated axons successfully penetrated the lesion site within 4 h after surgery.^122^

Neurogenesis in adult mammals mainly occurs in the subependymal area of the lateral wall of the ventricle and the subgranular area of the hippocampus. It has been proven that the spinal cord contains endogenous NSCs/NPCs within the ependymal cell population.^23,123–126^ After SCI, NSCs/NPCs are activated, migrate into the lesion site, and produce newborn astrocytes and oligodendrocytes.^124–129^ It was reported that the ependyma of the adult spinal cord is a latent stem cell niche that can be activated and contribute to glial scar formation.^124^ Interestingly, more effective endogenous regeneration was found in zebrafish after CNS injury, which was related to pluripotency via regulation of the key factors pou5f1 and sox2.^130^ In addition, connexin signaling in the ependyma changes after SCI, functionally resembling the immature active stem cell niche of neonatal animals, suggesting that connexins in ependymal cells are potential targets to improve self-repair of the spinal cord.^131^

#### Astrocytes

Astrocytes are the most abundant supporting cells of the nervous system. In response to SCI, astrocytes become activated and transform into reactive astrocytes, which protect the uninjured spinal cord from inflammatory cell infiltration and minimize initial damage at an early stage but later form a glial scar that is a physical barrier to nerve regeneration. There are two main subtypes of reactive astrocytes: neurotoxic astrocytes (A1 cells) induced by inflammation and neuroprotective astrocytes (A2 cells) induced by ischemia, and this functional transformation involves a variety of substances and intracellular signaling pathways.^132^ It was reported that astrocytes transform into A1 cells (with C3 as a marker) through the NF-κB pathway and into A2 cells (with S100A10 as a marker) through the STAT3 pathway.^133^ Recently, the Deneen group identified five subpopulations of astrocytes, named Populations A, B, C, D, and E, based on combinatorial expression of CD51/CD71/CD63 and found that population C possessed significantly enhanced synaptogenic properties in vitro. However, the astrocyte subpopulation distribution showed that Population A was dominant in the spinal cord in the presence or absence of injury. Therefore, subpopulation switching would be a promising repair target for SCI.^134^

Interestingly, microglia may be the most important cell type that triggers reactive astrogliosis. It was reported that A1 astrocytes are induced by three factors, Il-1α, TNFα, and C1q, which are all produced by activated microglia. The authors found that these three factors should be simultaneously present for LPS-induced in vitro polarization; if not, microglia do not induce astrocyte polarization.^135^ Additionally, type I collagen, which is expressed in the injured spinal cord, induces N-cadherin-mediated adhesion and is directly involved in the transformation of reactive astrocytes, as well as in astrocytic scar formation, after SCI.^136^

Astrocytes are neural parenchymal cells tile the whole mammalian CNS.^137^ The astrocytes provide multiple functions essential for the CNS functions, such as maintenance of the molecular, systemic and metabolic homeostasis,^138^ provision of metabolites to neurons,^139^ modulation of local blood flow,^140^ etc. In response to SCI, astrocytes exhibit morphological, molecular and functional changes, referred to as reactive astrocytes.^141–144^ Reactive astrocytes are highly heterogeneous, range from subtle and reversible alterations in gene expression and morphology to permanent astrocyte scar formation, depend on the distance from the injury and types of injury.^143,145,146^ Though reactive astrocytes were long regarded as functional passive, numerous newly researches provide evidences of their positive aspects,^146–148^ and may influence the outcome of SCI.^143^

Transcriptome analysis can divide reactive astrocytes into different clusters or subtypes according to their molecular signatures.^33,135,149,150^ For example, high-profile subtypes of A1 neurotoxic astrocytes and A2 neuroprotective astrocytes,^135,149^ which may represent new therapeutic potential. However, the function of these marker genes are not well known,^137^ and no experimental evidence proved the A1/A2 astrocytes marker genes exert either toxic or protective functions.^144^ Thus, the meaningful definition of reactive astrocytes subtypes should not solely based on the molecular signatures.^137^

#### Oligodendrocytes and OPCs

Oligodendrocyte apoptosis was detected in the white matter after 24 h and reached its highest level at 8 dpi.^151^ Oligodendrocytes may also have subtypes and different responses to SCI. In one study, six mature oligodendrocyte (MOL) subpopulations were described, and they presented different responses to SCI.^152^ During the acute phase, the responses of MOL2 and MOL5/6 to injury were similar, but during the chronic phase, the response of MOL2 at the injury site was decreased, while MOL5/6 reached a higher level.^152^ The overexpression of p53 can enhance the endoplasmic reticulum–mitochondria interaction and trigger the E2F1-mediated apoptosis pathway.^153^ However, it was also reported that inflammatory cells or their mediators had no significant destructive effect on oligodendrocytes during the early stage of SCI.^154^

Oligodendrocyte precursor cells (OPCs) are a subgroup accounting for 5–8% of the cells in the CNS, and they are a potential source of oligodendrocyte replacement after SCI.^155^ Traditionally, OPCs have been identified by their expression of NG2 and PDGFRα. After SCI, OPCs are activated, proliferate, differentiate into new oligodendrocytes and Schwann cells to regenerate axons, and participate in the formation of astrocyte scars.^156^

Endogenous OPCs have been proven to effectively and spontaneously repair the myelin sheath after SCI through genetic fate mapping.^157,158^ It was reported that OPCs (PDGFRα^+^) are responsible for in 30% of the new myelin sheath at the epicenter of SCI.^157^ However, the results showed that oligodendrocyte remyelination is not required for spontaneous recovery of stepping.^158^ This was contrary to the conclusions of other research.^159–162^

Regarding remyelination, the regenerative capacity of OPCs is conspicuously restricted by the hostile microenvironment of SCI, which includes factors such as scar-associated chondroitin sulfate proteoglycans (CSPGs)/microglial activation/Nrg-1.^163^ OPC proliferation and oligodendrocyte maturation following remyelination could be enhanced by neuronal activity and improved hindlimb motor function.^164^ In addition, it was shown that the proinflammatory reaction process is needed for the degradation of myelin debris and the generation of new oligodendrocytes.^165^

#### Interactions between immune cells and neural cells

As mentioned above, the immune microenvironment may indirectly lead to neural cell death. Meanwhile, the microglia-neuron interaction is an important factor in chronic pain after SCI. It was reported that the upstream regulator of prostaglandin E2 (PGE2) release, phosphorylated extracellular signal-regulated kinase 1/2 (pERK1/2), was specifically localized in microglia, while the PGE2 receptor E-prostanoid 2 (EP2) was localized in neuronal cells. Blocking the EP2 receptor resulted in a decrease in the hyperresponsiveness of dorsal horn neurons.^166^ In SCI in zebrafish, there is one macrophage subtype with high expression of TNFα that has direct communication with spinal progenitor cells and promotes neurogenesis.^58^

Microglia are essential for restoring tissue homeostasis and achieving optimal recovery after SCI. Microglia play these beneficial roles by regulating the transcriptional fate, function and intercellular crosstalk of various nonneuronal cell types.^167^ It was reported that microglia are indispensable in the process of astrocyte scarring.^75^ Reactive astrocytes interact with microglia within the glial scar via fibronectin, a major ligand of β1R, and enhance microglia-mediated immune inflammation.^168^

### Glial scar formation and function

The SCI lesion is composed of three main compartments: a nonneural lesion core, an astrocyte scar surrounding the lesion core, and spare but reactive neural tissue.^169–172^ The cellular components of these three compartments are quite different. Blood-borne cells such as fibroblasts and other immune cells leak from the disrupted blood‒brain barrier in the injured spinal cord. Local pericytes and fibroblasts also start proliferating. They produce extracellular matrix (ECM) components and form fibrotic scars, and almost no neural cells can be found in this toxic environment. Local astrocytes are activated by inflammatory reactions secondary to SCI and form a narrow astrocyte scar surrounding the fibrotic scar, protecting adjacent spare neural cells.^147,148,173,174^ In the distal area of the lesion, continuous with the astrocyte scar, the spare but reactive neural tissue contains neurons, astrocytes, oligodendrocytes, OPCs, and microglia.^172,175^ The features of this compartment include reactive astrocytes and OPCs with a hypertrophic cell morphology, and this area can be surprisingly large.^141^

#### Astrocyte scars

After SCI, resident astrocytes are activated by many molecules produced by all cell types in the spinal cord tissue.^176^ After mild injury, astrocytes upregulate GFAP, an intermediate filament protein, with hypertrophy of the cell body and processes, but preserve their original numbers without proliferation.^145^ After severe injury, astrocytes proliferate, migrate and organize around the severely damaged lesion center. They intertwine with their cell processes and form a dense scar tissue corral around the inflamed lesion center, which is named the astrocyte scar.^141,145,170,177,178^ The astrocyte scar is narrow, with only several cell layers separating the spare neural tissue from the nonneural lesion core, and its layers are continuous with the spare but reactive neural tissue.^141,171,177^ Experimental evidence revealed the beneficial aspects of the astrocyte scar. Astrocyte scars isolate and sequester the harmful lesion center from the neighboring spare neural tissue, which limits the lesion size to activate neuroprotection and regulate spinal cord homeostasis.^141,179^ After using GFAP-TK transgenic mice and ganciclovir administration to ablate dividing reactive astrocytes, failure of blood‒brain barrier repair, leukocyte infiltration, demyelination, neural cell death, and functional deficits were observed.^174^ In STAT3 conditional knockout transgenic mice, the astrocytic reaction after SCI was inhibited, and inflammatory cell infiltration increased, leading to worse functional outcomes.^180^ Additionally, no axon regeneration was observed when astrocyte scar formation was prevented or chronic astrocyte scars were ablated.^147^

Conversely, robust axon regeneration occurred despite the presence of an astrocyte scar under appropriate conditions (activation of neuron intrinsic growth capacity, growth supportive substrate and chemoattractive factors).^147^ The reason is that scar-forming astrocytes express laminin, an axon growth-supporting matrix protein, and laminin-integrin binding blockade attenuates axon regeneration.^147^ Axon growth-inhibitive CSPGs are produced by many cell types after SCI, and reactive astrocyte ablation cannot reduce CSPG levels. Scarce-forming astrocytes express numerous permissive molecules for axonal regeneration.^147^

Although there are also other cell types within the astrocyte scar compartment, such as OPCs, the mature astrocyte scar after SCI consists primarily of newly generated astrocytes.^177,181,182^

#### Fibrotic scars

After injury, the lesion center of the spinal cord undergoes hemorrhage, edema, etc. Blood-borne cells such as fibroblasts invade the spinal cord and secrete ECMs such as Type IV collagen, fibronectin, laminin and proteoglycan.^170,172^ Pericytes are also recruited by innate inflammation. They proliferate and form the fibrotic scar. Pericyte-derived cellular components of scar tissue are important for regaining tissue integrity. By using Glast-CreER transgenic mice and a Rosa26-YFP reporter mouse line, a subtype of pericytes named type A pericytes was labeled.^183^ Blocking the progeny of type A pericytes results in failure to seal the injured spinal cord.^183^ However, this kind of scar tissue is considered a barrier to axon regeneration.^184^ When a specific transgenic mouse line (Glast-CreERT2 Rasless, Rosa26-YFP) was used, the proliferation of type A pericytes was inhibited, and fibrotic scarring and ECM deposition were reduced. Enhanced axon regeneration and functional recovery were observed.^185^ Thus, due to the dual functions of the fibrotic scar, balancing the beneficial and detrimental effects of fibrotic scars is fundamental in treatment strategies targeting fibrotic scarring.

### Molecular mechanism of neural circuit damage

Primary SCI injury causes irreversible mechanical damage to the neural circuit, and subsequent axonal disruption, degeneration, demyelination, and neuronal death lead to more severe neurological dysfunction. Local injury of the spinal cord causes changes in the sensitivity and excitability of neurons, which may lead to pathological pain and even cause neurodegeneration of the spinal cord remotely from the injury site.

#### Acute phase

During the acute stage of SCI, the excitability of sensory and motor neural circuits is altered, in addition to mechanical damage to the spinal cord, which may be the pathophysiological mechanism of spinal cord concussion or spinal cord shock in SCI patients. One study that dynamically detected neural circuit changes in lamprey with SCI showed that spinal cord excitability was significantly reduced above and below the lesion site, and excitatory synaptic inputs to motor neurons recovered earlier than those to sensory neurons.^186^ Interestingly, the change in interneuron excitability in spinal cord tissue was related to functional recovery after SCI.^187,188^ It was reported that neonatal mice with SCI showed spontaneous recovery because they maintained the excitatory phenotype of glutamatergic interneurons, with the induction of synaptic sprouting to facilitate excitation. In contrast, SCI in adult mice promotes neurotransmitter switching of spatially defined excitatory interneurons to an inhibitory phenotype.^188^ The excitability of spinal cord inhibitory interneurons may be the crucial factor limiting the integration of descending inputs into relay circuits after SCI.^187^

#### Chronic phase

Chronic phase changes in the spinal cord neural circuit are mainly reflected by functional remodeling based on compensatory mechanisms in the brain or spinal cord, including cortical compensation mechanisms and synaptic plasticity through spared axonal sprouting.^189–191^ The spared tissue and spontaneous repair of the corticospinal tract mediated by interneurons combine to form a new neural circuit and are an important basis for rehabilitative treatment.^192,193^

During the chronic stage of SCI, corresponding degenerative changes are present in the injured spinal cord^194,195^ and even the brain.^196^^,^^197^ Yokota et al. used a complete SCI model and found that atrophic changes were widely observed in the injured spinal cord both rostral and caudal to the lesion, but the decrease in area was mainly in the white matter in the rostral spinal cord, while both the white and gray matter showed a decreased area in the caudal spinal cord. However, the motor neurons in the caudal part of the injured spinal cord showed good potential for synaptogenesis, with high expression of acetylcholine-related molecules.^195^ Azzarito et al. used quantitative MRI and found that in patients with SCI, the cord area and left-right width of the remote cervical spinal cord were decreased, and atrophy of the cerebral cortex was sustained when spinal cord atrophy became slower. The degree of atrophy of the spinal cord and corticospinal tract at 6 months after SCI was closely related to motor function recovery at the 2-year follow-up.^196^

SCI can induce chronic neuropathic pain, cognitive deficits and physiological depression, which may be involved in chronic inflammation of the brain through sustained induction of M1-type microglia.^198^ It has also been reported that CNS injury can trigger APP and Tau cleavage by delta-secretase (AEP) and mediate Alzheimer’s disease pathology.^197^

Sequential study on pathological mechanism of SCI provides intervention targets for sequential treatment.^33,199,200^ In general, the acute stage of SCI is dominated by neuroprotection and neuroinflammatory regulation, including the use of neuroprotective drugs, reducing the infiltration of inflammatory cells, and reducing oxidative stress; In subacute stage, it mainly regulates scar formation, angiogenesis and promotes nerve regeneration, including materials, cell transplantation and the application of small molecular compounds; In the chronic phase, the compensatory recovery of neural function can be promoted by means of physical regulation, the neural circuit can also be reconstructed by removing glial scar, combining biomaterials and/or stem cells, and astrocytes can even be transdifferentiated into neurons by means of reverse transcription.^35,201,202^ It is worth mentioning that neuroimmunity participates in the whole process of SCI pathology, especially the role of microglia. We have previously confirmed that the immune response is negatively related to nerve regeneration, and the immune-deficient mice show better neural function recovery.^28^

## Intervention and repair of SCI

**Fig. 3:**
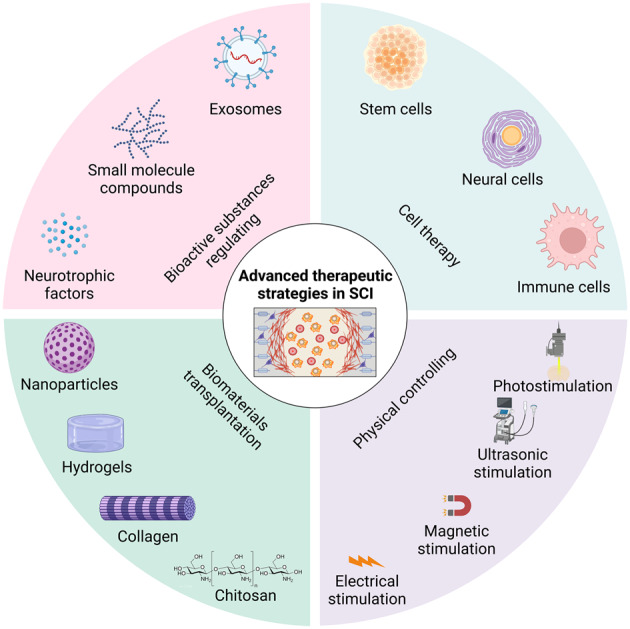
Schematic depiction of various advanced therapeutic strategies for repairing SCI based on the studies. Strategies including bioactive substances regulating, cell therapy, biomaterials transplantation, and physical controlling, are applied to repair SCI from different perspectives. Meanwhile, the combined use of these strategies has also received increasing attention from researchers. The figure is generated from BioRender.com

A spontaneous repair mechanism exists after SCI, but it faces many difficulties and external intervention is needed to further improve the repair ability. Small biological molecules can provide nutritional factors for neural regeneration or regulate cell metabolism.^203^ Stem cells can effectively differentiate to replace apoptotic neural cells. Bioactive materials and physical regulation approaches can regulate cell fate at the site of SCI (Fig. 3). These approaches can promote the generation of newborn neurons and the formation of intermediate neural network, which is conducive to the function of SCI.^34,204^

### Bioactive substances

At present, bioactive substances such as neurotrophic factors, small-molecule compounds, and exosomes are widely used in SCI research, and they all show the ability to promote functional recovery after SCI through axon regeneration and microenvironment improvement.^205^ Traditional Chinese medicines, such as ginsenosides, genistein and tanshinone, also mediate neuroprotection and promote neural function recovery of SCI.^206–209^

#### Neurotrophic factors

The roles of different neurotrophic factors vary. Brain-derived neurotrophic factor (BDNF) has functions in axonal regeneration, neurogenesis protection, remyelination, synaptic reformation and synaptic transmission in different neuronal populations after SCI.^210^ Neurotrophin-3 (NT⁃3) has recently received substantial attention because it can promote oligodendrocyte proliferation and neuronal survival and does not cause side effects such as pain or cramping.^211^ In addition, many studies have demonstrated that NT3 plays a key role in promoting neural circuit remodeling.^23,34,212^ Ciliary neurotrophic factor (CNTF) has been proven to promote neuronal development and increase the survival rate of severed axons.^213^ Fibroblast growth factor (FGF) is involved in stimulating axonal growth, promoting angiogenesis, and exerting anti-inflammatory and neuroprotective effects in inflammatory cells.^214^ Glial cell-derived neurotrophic factor (GDNF) can promote axonal regeneration in the CNS and PNS after SCI.^215^ A new factor called nerve growth factor inducible (VGF) was recently identified by our team, and we found that VGF-mediated oligodendrogenesis could benefit SCI repair.^216^ Although the use of such factors is very promising, there are disadvantages, such as unstable physicochemical properties and high costs, that severely limit their application and remain to be addressed.

#### Small-molecule compounds

Small-molecule compounds have the unique advantages of high cell permeability, reversibility and ease of manipulating cell fate regulation; thus, this approach is a promising new strategy to regulate cell fate. It was reported that minocycline can reduce the volume of necrotic tissue after SCI and improve the motor function score in animals.^217,218^ A selective type 2 lysophosphatidic acid receptor (LPA2) antagonist was reported to effectively improve the inflammatory microenvironment after mouse SCI via lipid metabolism regulation.^219^ It was also reported that the potassium-chloride cotransporter-2 (KCC2) agonist CLP290 can restore stepping ability in paralyzed mice by reducing the excitability of spinal cord inhibitory interneurons.^187^ An interesting study showed that small-molecule peptides could mediate intercellular signal transmission, enhance supramolecular movement, and activate signaling pathways related to neural regeneration and repair after SCI.^220^

#### Exosomes

Exosomes (Exos) have been gradually found to play an important role in signal transmission in various physiological and pathological states, including SCI. Exos are vesicles with a diameter of approximately 40–120 nm that are continuously released into the extracellular environment by cells.^221^ They are formed by endosomes resulting from membrane endocytosis, which bud into the lumen to form multiple vesicles, and then the multiple vesicles fuse with the membrane and are released into the extracellular matrix. Therefore, exosomes contain large numbers and different kinds of proteins, lipids, RNAs and other biologically active factors.^222,223^ Exosomes can effectively promote functional recovery after SCI through their immunomodulatory, anti-inflammatory, and anti-apoptotic effects as well as their ability to promote vascular and axon regeneration. Meng et al. proved the presence of large amounts of granulocyte–macrophage colony-stimulating factor (GM-CSF) in exosomes, which have the potential to enhance immunomodulatory function and benefit SCI repair.^224^ Zhou et al. showed that anti-inflammatory microglia-derived M2-Exos had a better ability to promote the recovery of functional behavior, increasing axon regeneration and reducing the level of pyroptosis in spinal cord neurons after SCI. M2-Exos rich in miR-672-5p could inhibit the AIM2/ASC/caspase-1 signaling pathway by inhibiting AIM2 activity to inhibit neuronal pyroptosis and ultimately promote the recovery of functional behavior in mice with SCI.^225^ Pan et al. revealed that Schwann cell-derived exosomes can promote functional recovery of mice post SCI by decreasing CSPG deposition via an increase in TLR2 expression on astrocytes through the NF-kappaB/PI3K signaling pathway.^226^

### Advanced cell therapy for SCI repair and regeneration

Mature neurons are difficult to regenerate after damage, and it is crucial to find an ideal, simple, safe, effective and feasible repair strategy to promote axonal regeneration, remyelination and functional recovery. Cell transplantation has emerged as the most promising therapeutic approach for SCI. Cells transplanted into the site of SCI have the potential to differentiate, secrete a variety of cytokines and growth factors, regulate the inflammatory response, provide nutritional support, and promote axonal regeneration and nerve repair. Direct or indirect interactions between transplanted and host cells respond to the microenvironment at the site of spinal cord damage and are also able to influence the microenvironment at the lesion site in the spinal cord and alter the interactions between transplanted and host cells, thus affecting tissue and functional outcomes after SCI.

#### Stem cells

Stem cells are a class of multipotent cells with self-replication and differentiation abilities; they play important roles in tissue repair and regeneration, and they are expected to be important therapeutic tools for neurological diseases as seed cells. In recent years, stem cell transplantation has been widely used in SCI repair, and a variety of stem cells have been applied in clinical practice. Stem cells can differentiate into neural precursor cells, oligodendrocytes, astrocytes, and neurons, which can promote axonal regeneration, bridge the diseased lumen, and promote functional recovery by replacing missing cells or regulating the microenvironment at the site of injury.

##### Embryonic stem cells

Embryonic stem cells (ESCs) are derived from endocytic clusters in the blastocyst stage, have a high differentiation potential, and can be induced to differentiate into almost any cell type. If transplanted in undifferentiated form, they are prone to form teratomas in vivo, which severely limit the application of ESCs. Currently, ESCs are generally differentiated into specific cell types, such as neural precursor cells, specific neurons or glial lineages, and then transplanted.

Human ESC-derived neural crest cells can promote remodeling of descending raphespinal projections and contribute to the partial recovery of forelimb motor function in SCI animal models.^227^ Kim et al. evaluated the efficacy and safety of human polysialylated neural cell adhesion molecule (PSA-NCAM)-positive neural precursor cells (hNPCs (PSA-NCAM^+^)) as a treatment for SCI.^204^ hNPCs (PSA-NCAM^+^) differentiated into neural cells and successfully integrated into the host tissue with no evidence of tumor formation, which also significantly improved locomotor function.

##### Induced pluripotent stem cells

Induced pluripotent stem cells (iPSCs) are obtained by reprogramming of genes such as Oct3/4, Sox2, Klf4, and c⁃Myc via transduction into mouse or human fibroblasts and are expected to be the preferred cell source for human SCI therapy because of ethical issues.

Gong et al. showed that spinal GABA interneurons efficiently generated from iPSCs could form synapses with host spinal neurons and mitigate the spasticity-like response of the rat hindlimbs and locomotion deficits within 3 months.^228^ Wertheim et al. reported an approach to recapitulate the embryonic development of the spinal cord by using iPSCs, which were further encapsulated in ECM-based hydrogels, and the implants enriched the targeted region with biochemical and mechanical cues to attract progenitor cells, supported cell survival and engraftment, reduced inflammation and gliosis at the lesion site, and overall improved the locomotion of the treated animals.^229^

##### Neural stem cells/neural progenitor cells

NSCs/NPCs are pluripotent stem cells with self-renewal ability that are able to differentiate into neurons, astrocytes and oligodendrocytes and can replace damaged cells at the injury site and secrete a variety of neurotrophic molecules. NSCs/NPCs have the ability to reduce cell death, reduce lesion volume, inhibit scar formation, exert anti-inflammatory effects, and promote electrophysiological and motor function recovery.^26,230,231^ They have advantages in forming effective neural networks at the injured site.^232–235^ For NSCs/NPCs, there are two different strategies, namely, transplantation of exogenous NSCs and activation of endogenous NSCs.

For exogenous NSC transplantation, many studies have applied materials for better cell transplantation. For example, Zou et al. proved that collagen sponge-based 3D-cultured NSCs cultured in a rotary cell culture system had better therapeutic effects than those cultured in a traditional cell culture environment, and this novel and effective method shows promise for application in NSC-based therapy for SCI.^236^ Liu et al. showed that collagen scaffolds combined with each type of NSC could markedly restore the motor function of the hindlimbs, as indicated by Basso-Beattie-Bresnahan (BBB) scoring, and further proposed that allogeneic NSC transplantation promotes functional recovery after SCI predominantly via the secretion of neurotrophic factors, not via direct neuronal replacement with neurons differentiated from transplanted cells.^237^

For the activation of endogenous NSCs, many methods, including biomaterials, pharmaceuticals, and electrical stimulation, have been applied. Zhu et al. demonstrated the in vivo behavior of LDH nanoparticles and LDH/NT3 in mice with complete spinal cord transection, both of which could contribute to the proliferation and differentiation of endogenous NSCs, reduce the inflammatory response at the injured site and improve the microenvironment to promote regeneration. These findings support an immunomodulatory strategy to recruit native NSCs as a potential acute care intervention for SCI.^34^ In this study, a combination treatment with pioglitazone (PGZ) and granulocyte colony-stimulating factor (GCSF) was applied in a rat T9 contusion model of SCI, and PGZ and GCSF treatment synergistically enhanced NSC numbers and improved functional recovery after SCI, which proved that this treatment can support NSCs directly and provide a sustainable microenvironment.^22^ Electrical stimulation has generated promising evidence as a novel approach to activate NSCs to facilitate neural repair, and more recently, clinically focused therapies aimed at improving outcomes following SCI have investigated the application of epidural electric stimulation. To date, this has been proven to be a promising rehabilitation strategy when used in conjunction with physiotherapy.^21,238^

##### Mesenchymal stem cells

Mesenchymal stem cells (MSCs) are obtained from a wide range of sources, such as bone (BMSCs), adipose tissue (AT-MSCs), umbilical cord (UCMSCs), and dental pulp (DP-MSCs). They are pluripotent stem cells that can self-renew and directionally differentiate into other types of cells^239,240^ and have low immunogenicity and multiple differentiation potential, making them popular in the stem cell and regenerative medicine fields.^241–245^ These cells can secrete colony-stimulating factor, stem cell factor, nerve growth factor, and other cytokines.^246^ In terms of promoting neuronal regeneration and restoring neuronal pathways, MSCs have obvious advantages in regulating the injury microenvironment and providing neurotrophic factors.^221,247,248^

After transplantation in the SCI model, MSCs mainly protect neurons in the following ways. First, MSCs play an immunomodulatory role in the microenvironment by inhibiting inflammation. MSC transplantation can inhibit the activities of various inflammatory factors (IL-1α, IL-1β, and TNF-α) and inflammatory cells (T cells, B cells, and macrophages) and reduce the inflammatory response in the lesion area after SCI. Studies have shown that transplantation of MSCs into SCI rat contusion models can significantly increase the number of M2 macrophages and decrease the number of M1 macrophages at the injury site, which might contribute to the recovery of motor function, increased retention of axons and myelin sheaths and reduced glial scar formation after injury.^249^ Second, MSCs secrete a variety of neurotrophic factors, such as BDNF, NT3, NGF, and GDNF, to play a neuroprotective role.^250^ On the other hand, MSCs can also act on T lymphocytes, B lymphocytes, natural killer cells (NK cells), antigen-presenting cells and other immune cells in various ways to affect the immune state of the body, reduce the immune response of the body and promote the repair of SCI by inhibiting their proliferation, differentiation and activation.^251^ Recently, it was reported that the mechanism by which BMSCs reduce neuronal apoptosis after SCI may involve the transfer of mitochondria to neurons via gap junctions.^252^

##### Oligodendrocyte progenitor cells

Oligodendrocyte progenitor cells (OPCs) are adult stem cells widely distributed in the central nervous system. As precursor cells of oligodendrocytes (OLs), OPCs can migrate to affected areas and differentiate into OLs under the action of a variety of chemokines to promote the formation and regeneration of the myelin sheath. They are beneficial to the repair of demyelinating lesions. Results have shown that OPCs can survive in the spinal cord of rats with SCI after transplantation and differentiate into neurons such as OLs and astrocytes, which promote myelination of the injured site, repair the damaged spinal cord tissue, and improve the motor ability and evoked potential generation of rats.^253^ It was reported that when human EMC-derived OPCs were transplanted into the cervical spinal cord 1 week after injury in rats, they significantly improved locomotor performance with no adverse clinical effects.^254^

#### Neural cells

##### Olfactory ensheathing cells

Olfactory ensheathing cells (OECs) exist in both the peripheral nervous system and central nervous system, have regeneration ability, and are unique glial cells that show promise for the treatment of SCI.^255^ Barbour et al. found that OEC transplantation significantly increased neuronal survival by approximately sixfold in rats with SCI (T10) in the subacute stage.^256^ Combined transplantation of OECs and NSCs in rats with SCI showed that OECs could guide axonal lengthening through the glial scar and promote myelination. A key ability of OECs is migration from the peripheral nerve to the central nervous system, which enables the enhancement of axon extension after SCI and contributes to nerve regeneration.^257^

##### Schwann cells

Schwann cells (SCs) act as structural scaffolds for the peripheral nervous system and can promote a microenvironment favorable to neuronal regeneration.^258^ In the central nervous system, they can regenerate demyelinated axons by secreting a variety of growth factors and depositing growth-promoting proteins in the extracellular stroma. Barbour et al. performed local injection and transplantation of SCs in an acute (14 d after injury) rat model of moderate SCI and observed that the SC cell injection group showed an increased number of supraspinal fibers, a reduced appearance of cavities and enhanced tissue integrity 4 months later, indicating improved anatomical outcomes after SCI.^256^ Autologous activated Schwann cell (ASC) transplantation for the treatment of SCI was carried out in China ten years ago and showed some signs of functional improvement.^259^

##### Astrocyte lineage

Astrocyte transplantation was thought to be an important strategy for SCI repair because of the formation of local cavities after SCI and the important supporting role of astrocytes in the regeneration and extension of neuronal axons.^260^ Lepore et al. transplanted cultured lineage-restricted astrocyte progenitors into animals with cervical SCI and found that these cells could survive for a long time and differentiate into astrocytes, promote motor neuron axon regeneration, and improve diaphragm function.^261^

#### Immune cells

##### Macrophages

The proportion of proinflammatory/anti-inflammatory immune cells at the SCI site can be adjusted by transplanting macrophages with immunomodulatory effects. It was reported that transplantation of M2-deviated microglia induced by IL-4 could improve the recovery of motor function in mouse SCI.^70^ In addition, M2-phenotype (M2) macrophages induced by tauroursodeoxycholic acid had a similar effect.^201^

### Advanced biomaterials for SCI repair and regeneration

In recent years, nanotechnology has made great advancements in the treatment of SCI. Nanomaterials can be used as nanocarriers for targeted drug delivery, and increasing the cycle time can improve the bioavailability of drugs. Recently, many biomaterials have been designed and have shown advantages in eNSC activation, mobilization, and controlled differentiation.^34,262–266^ Some microenvironment-responsive biomaterials have shown good immunoregulatory effects.^267^ In research on the repair and regeneration of SCI, a number of natural and composite materials have been utilized, including nanoparticles, hyaluronic acid, alginate, collagen, agarose, polylactic acid, PLGA, etc. The key issues that need to be addressed are to reduce inflammatory infiltration, reduce scar tissue, improve the regenerative capacity of neurons and axons, and guide the axons to the appropriate areas for regeneration.^268^ Here, we focus on several biomaterials with clinical application prospects.

#### Inorganic-layered nanomaterials

**Fig. 4:**
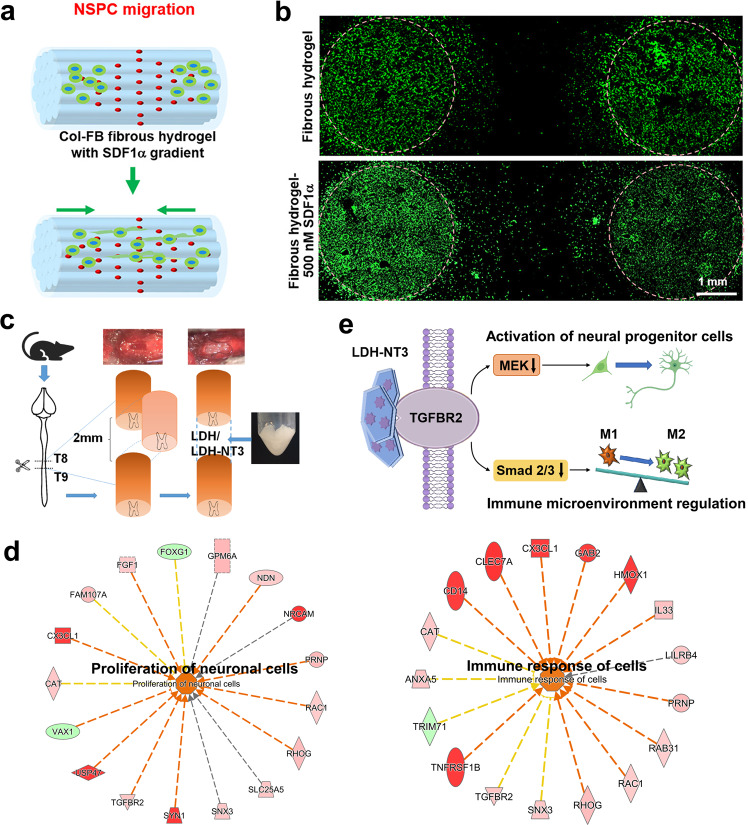
Advanced biomaterials for bioactive molecule delivery and microenvironment regulation. **a**, **b** Functionalized aligned Col-FB fibrous hydrogel induced NSPC migration and neuronal differentiation. Adapted with permission from ref. ^287^ Copyright 2022, American Chemical Society. **c**–**e** The LDH/LDH-NT3 transplantation promoted the process of neural regeneration and neural circuit reconstruction in the lesion sites of SCI mice. Adapted with permission from ref. ^34^ Copyright 2021, American Chemical Society

Nanoparticles are increasingly being studied in experimental models for SCI treatment. The composition of these nanoparticles is extremely diverse and includes polymers, metals, metal oxides, silica, and biological molecules.^269–271^ In our previous study, we used biodegradable Mg/Al LDH as a novel strategy for immune microenvironment amelioration and neural regeneration. The results indicated that both LDH and LDH-NT3 could improve the microenvironment to accelerate NSC migration, neural differentiation, L-Ca^2+^ channel activation, and inducible action potential generation, which supported the generation of newborn eNSCs at the lesion site.^34^ In addition, LDH-NT3 performed remarkably well in regulating synaptic transmission and neuron–neuron synaptic transmission. With the improved microenvironment established by LDH/LDH-NT3, neural precursor cell synthesis, axonogenesis and ion channel action-involved signaling pathways were positively regulated to achieve regeneration of neurons and the reconstruction of neural circuits after SCI (Fig. 4).^34^ Graphene and graphene-based materials have good electrical conductivity, which can make full use of nerve electrical signals in spinal cord tissue to promote axon regeneration.^272,273^

#### Hydrogels

As a large category of biological materials, hydrogels can mimic soft tissue environments and have suitable chemical compositions for the integration of extracellular matrix (ECM) molecules and other binding proteins, which can effectively support and guide axon regeneration for SCI repair.^34,274–278^ Zaviskova et al. modified the hydroxy groups of hyaluronic acid with RGD phenyl derivatives and crosslinked them with enzymes to obtain soft injectable hydrogels.^271,279–281^ The obtained hydrogel and hydrogel+MSCs were transplanted for the treatment of SCI, which showed strong effects on axon growth at the center of the injury when used in combination. Mukhamedshina et al. used fibronectin-based hydrogels for the culture and transplantation of ADSCs in SCI, and the results showed that the expression of GFAP and Iba1 decreased, with a smaller area of the center cavity.^282–285^ Dai et al. reported a microenvironment-responsive hydrogel that can effectively inhibit MMP and release the loaded bFGF according to the needs of the SCI microenvironment.^286^ The author’s other research revealed that one aligned collagen-fibrin (Col-FB) fibrous hydrogels showed good stretchable properties, adhesive behavior, and spatiotemporal delivery capability, and could promote locomotion recovery through recruiting endogenous neural stem/progenitor cells (Fig. 4).^287^

#### Collagen

Due to its low immunogenicity, good biocompatibility and biodegradability, appropriate porosity and mechanical strength, collagen has been proven to be one of the most suitable natural polymer materials for SCI repair. SCI triggers a biochemical cascade that creates a microenvironment around the injury site, which inhibits further nerve regeneration. Several signaling molecules in this microenvironment have been found by various scientists to inhibit neuronal axon regeneration. Dai et al. found signaling molecules in the SCI microenvironment that inhibit the differentiation of neural stem cells into neurons, and they have been trying to reconstruct the spinal cord regeneration microenvironment through functional biomaterials for many years. They established rat and beagle models of complete transection SCI to systematically study the effects of biological materials on regeneration in the SCI microenvironment.^288–291^ Functional biomaterials can reconstitute a regenerative microenvironment and antagonize the inhibitory effects of myelin protein on neural regeneration, thus inducing the differentiation of endogenous or transplanted neural stem cells into neurons. These new neurons form neural bridges through the injury area and transmit neural signals to promote the recovery of nerve function in animals with transection SCI. Research suggests that neural bridges formed by neurons generated from endogenous or exogenous neural stem cells are the main mechanism underlying functional biomaterial-based repair of complete transection SCI.^269,292–296^

#### Chitosan scaffolds

Yang et al. implanted an NT3-coupled chitosan biomaterial into a 5 mm space in the thoracic segment of rats in an SCI model, and eNSCs were activated in the injured spinal cord.^297^ Li et al. prepared a chitosan/ECM/SB216763 scaffold for SCI repair, and after transplanting chitosan/NT3 composites in a rat spinal cord T9 complete transection defect model using chitosan as a substrate, NeuN- and Tuj1-positive neurons appeared in the spinal cord defect area, with effective synaptic connections between neurons, resulting in significant recovery of hindlimb motor function in rats.^298^ In vivo experiments were conducted in a semitransection SCI model, and the results showed that the BBB score of the chitosan/ECM/SB216763 group was significantly better than those of the other groups.^299^

### Physical regulation and regeneration

#### Light stimulation

Optogenetics can play a very important role in rebuilding lost neuronal circuits. Ahmad et al. first reported that ChR2 could be expressed in motor neurons or stem cells, thereby stimulating neuronal activation and regeneration, in response to blue light irradiation.^300^ The p42/p44-MAPK signaling pathway can be modulated optogenetically to counteract the antagonistic effect of p38 MAPK, which can regulate long-term potentiation (LTP) of the mammalian hippocampus.^300–302^ Another noninvasive method, called photobiomodulation (PBM), can promote functional recovery by reducing neuroinflammation and promoting neuronal axon regeneration after SCI.^303^ It was reported that photobiomodulation is useful in polarizing macrophages via the NF-kB P65 pathway.^304^ Neurotoxic microglia and astrocytes atcivation is inhibited by PBM through Lcn2/JAK2-STAT3 crosstalk suppression.^305^ Additional investigations indicated that PBM therapy at 810 nm upregulates macrophage secretion of neurotrophic factors via PKA-CREB and promote neuronal axon regeneration in vitro, and the activation and secretory function of astrocytes were inhibited by photobiomodulation via alterations in macrophage polarization.^306^

#### Ultrasound stimulation

Ultrasound can regulate the proliferation and differentiation of stem cells. The first successful attempt in 1958 revealed that ultrasound could reversibly regulate nerve signal transmission and regulate central nervous system circuits in cats.^307^ Sangjin et al. showed that focused ultrasound excites primary murine cortical neurons in culture through a primarily mechanical mechanism mediated by specific calcium-selective mechanosensitive ion channels.^308^ Liao’s study indicated that low-intensity focused ultrasound (LIFU) can alleviate spasticity following SCI by activating spinal neurocircuits and increasing the expression of the neuronal K-Cl cotransporter KCC2.^39^ A proteomics study analyzed the effect of LIFU on spasticity post SCI and showed that Gap43 protein expression was significantly decreased in the LIFU therapy group.^309^ Recent works provide a mechanistic explanation for the effect of ultrasound on neurons to facilitate the further development of ultrasonic neuromodulation and sonogenetics as tools for neuroscience research.

#### Magnetic stimulation

A pulsed magnetic field was used to treat cultured fetal rat dorsal root ganglia in vitro, and it could induce electric fields and promote the growth of axons along the current direction.^310^ Low-frequency magnetic fields have better potential for promoting neuron proliferation and differentiation and neural circuit remodeling, but the specific parameters are not yet clear.^311^ It was reported that 50 Hz could affect neural excitability by regulating cortical calcium channels through the AA/LTE4 signaling pathway.^312^ Xue et al. also found that 50 Hz can enhance the expression of calcium channels on the presynaptic membrane of the mouse brainstem, thereby enhancing the calcium current and promoting the circulation of presynaptic vesicles and synapse touch plasticity.^313^ The effect of iron oxide nanoparticles (IONPs) along with electromagnetic field (MF) exposure on spontaneous axonal sprouting after SCI was evaluated. Under exposure to MFs (50 Hz, 17.96 μT, and 2 h/day for 5 weeks), the results showed sprouting from mature neurons and axons, significantly less demyelination and more myelinated fibers at the lesion site.^314^ A mild contusion rat model of SCI suggested that facilitation of sensory-motor recovery occurred after MF exposure, which could be due to attenuation of secondary damage and calcium-mediated excitotoxicity.^315^

#### Electric stimulation

The polarity characteristics of electrical stimulation have regulatory effects on the migration and differentiation of stem cells.^316,317^ It was reported that pulsed DC stimulation (1 V/cm for 12 days) is most effective in enhancing the differentiation of NSCs into neurons.^318^ Petrella et al. compared the effects of a picosecond pulsed electric field on NSCs and MSCs. Pulsed ES has no influence on MSCs proliferation but improves NSCs proliferation and astrocyte-specific differentiation by upregulating GFAP after 24 h at 40 kV/cm.^319^ Dong et al. stimulated NSCs with electricity for 3 days at 150 mV/mm, resulting in increased achaete-scute homolog (Ascl1) expression that was further proven to regulate the phosphatidylinositol 3-kinase/protein kinase B (PI3K/Akt) pathway in NSCs.^320^ To date, the effects of electrical stimulation through 2D or 3D conductive materials on stem cell fate have been thoroughly investigated. Nanopatterned polyurethane-acrylate substrate surfaces and PLGA/GO composite membranes were effective in promoting the proliferation, differentiation and neurite elongation of NSCs.^321,322^ Stem cells showed improved cell behavior in the 3D culture system. Higher neuronal gene expression levels were observed, and more stem cells differentiated into neural cells with electrical stimulation. The underlying mechanism may be due to the upregulation of neural genes, such as MAP2, βIII-tubulin, and NSE, by electrical stimulation.^323,324^

The more mature application of electrical stimulation for SCI rehabilitation is functional electrical stimulation technology, whose mode of action is determined by three parameters: pulse amplitude, pulse duration and pulse frequency. The noninvasive nature of this approach makes it possible to use FES very early in the rehabilitation of patients who have had an SCI.

## Clinical treatment and research

At present, the clinical treatment methods for SCI include medications, surgery, rehabilitation and nursing. Management in the acute phase aims mainly to stabilize the condition and ensure the survival of patients. Management in the chronic phase aims mainly to restore function, reduce complications, and encourage patients to return to society and work. During this period, it may also be necessary for psychologists to treat patients with psychological disorders. Breakthroughs in clinical treatment have mainly focused on the research and development of new drugs, clinical trials of cell therapies and biomaterial transplantation, new physical regulation approaches, artificial intelligence, etc.

The pathophysiological mechanisms driving the secondary injury after SCI are complex and SCI is a heterogeneous condition.^27,33,325^ Single treatment may only affect a small portion of mechanisms, whereas combinatorial treatment targeting multiple mechanisms is potentially a better therapeutic selection. In animal studies, while provide a combination of essential factors for axon regeneration-permissive substrates, chemoattractive growth factors and activating intrinsic growth capacity, robust axon regeneration through the astrocytic scar and nonneural lesion core after complete SCI was observed.^8,326^ Regeneration can also be accentuated by combining cells with biomaterials or neurotrophic factors.^327^ For instance, Schwann cells combined with neurotrophins, elevation of cyclic AMP levels, olfactory ensheathing cells, a steroid or chondroitinase,^328^ NSCs expressing embedded into fibrin matrices containing growth factor cocktails,^235^ combination of Schwann cells, OECs chondroitinase,^329^ NSCs, fibrin matrices and a cocktail of growth factors and a cell death inhibitor, etc.^330^ A speculative pharmaceutical cocktail of three commercially available agents including thyrotrophin-releasing hormone, selenium and vitamin E for intravenous and oral administration was proposed as a combination of medications to treat acute SCI based on theoretical benefits such as antagonism of endogenous opioids, petidoleukotrienes, excitoxins, and antioxidant properties. However, no animal or human studies of such cocktails were published, thus this proposal may contentious.^331^

### Clinical treatment

#### Drugs

Methylprednisolone (MP) is the only FDA-approved drug for the treatment of SCI. The mechanism of MP in SCI mainly involves reducing the secondary inflammatory response, restoring the blood‒spinal cord barrier, improving the spinal cord blood supply, scavenging free radicals, and enhancing neurotrophic factor secretion.^332–335^ However, recent studies did not recommend the use of MP for the treatment of acute SCI because of substantial clinical evidence for deleterious side effects of MP in acute SCI.^336^ The latest AO guidelines for SCI recommend 24 h infusion of methylprednisolone only in SCI patients within 8 h of injury but not in patients more than 8 h from injury.^337^

Clinical trials of new drugs for SCI are constantly being carried out. These studies have focused on neural regeneration and microenvironmental regulation after SCI, including studies of glyburide (NCT02524379), buspirone combined with levodopa-carbidopa (NCT04052776), and vitamin D3 (NCT04400747). Research institutes have also given attention to the drug treatment of SCI-related complications, such as neuropathic pain (Lyrica, NCT00879021; Controlled-Release Morphine, NCT00488969; GW-1000-02, NCT01606202; Amitriptyline, NCT00006428; KAI-1678, NCT01135108), neurogenic bladder (Neostigmine and Glycopyrrolate, NCT02370862; Botox-A, NCT00711087, Fampridine-SR, NCT00041717), and osteoporosis (Alendronate, NCT02195895; Teriparatide, NCT02025179), and a series of clinical trials of drug therapies have achieved some clear clinical effects (Table 2).

**Table 2:** clinical trials about drug therapy for SCI

Drugs	Patients condition	Frequency of administration	Study types	Subjects	Phase	ClinicalTrials.gov identifier or refs
Glyburide	Acute SCI	3.125 mg po on day 1, 2.5 mg for following days to 2 weeks	Interventional	3	Phase 1/2	NCT02524379
Buspirone and Levodopa-Carbidopa	SCI with EES	40 mg Buspirone, 400 mg/100 mg Levodopa-Carbidopa po. single-dose administration	Interventional	8	Phase 1	NCT04052776
Vitamin D3	Thoracic-level chronic SCI	6000 IU per day or 50,000 IU per week for 8 weeks	Interventional	60	Not applicable	NCT04400747
Lyrica	Traumatic SCI	150 mg p.o. Bid for 49 weeks	Interventional	5	Phase 3	NCT00879021
Modified-release morphine	Traumatic SCI	up to 120 mg p.o. for 7 weeks	Interventional	17	Phase 2	NCT00488969
GW-1000-02	Non-acute SCI	100 μl (THC 2.7 mg and CBD 2.5 mg)/time 8 times in 3 h, and 48 times in 24 h, 7–21 days.	Interventional	116	Phase 3	NCT01606202
Amitriptyline	6 months post SCI	N/A daily dose for 6 weeks	Interventional	100	Phase 4	NCT00006428
KAI-1678	1 year post SCI	N/A dose KAI-1678 i.v.	Interventional	5	Phase 2	NCT01135108
Neostigmine and Glycopyrrolate	SCI	Visit 1: 0.03 mg/kg NEO and 0.006 mg/kg GLY,i.v. Visit 2: 0.05 mg/kg NEO and 0.01 mg/kg GLY, i.v. Visit 3: 0.07 mg/kg NEO and 0.14 mg/kg GLY, i.v. 2–14 days.	Interventional	28	Phase 1	NCT02370862
BOTOX-A	T10 or above Thoracic-Level SCI 8 weeks post injury	100 units BTX-A injections on day 0 and day 90	Interventional	1	Phase 2	NCT00711087
Fampridine-SR	18 months post traumatic SCI	25 mg p.o. bid, 12 weeks	Interventional	213	Phase 3	NCT00041717
Alendronate	Chronic SCI	70 mg p.o. weekly for 12 months	Interventional	17	Phase 2	NCT02195895
Teriparatide	Chronic SCI	20 ug daily Sub-Q over 12 months	Interventional	25	Phase 2	NCT02025179

Riluzole is known as a neuroprotective agent.^5^ As a sodium channel blocker, Riluzole blocks the sodium channels and prevents the excessive influx of sodium ions, reduces the intracellular sodium concentration and influx of calcium ions which cause the development of intracellular acidosis and cytotoxic edema.^338^ In animal studies, riluzole provides histological and functional recovery.^339–342^ In a prospective, multicenter phase I trial, compared with the control group, the mean motor score showed significant improvement in the riluzole-treated group, and there were no serious adverse events related to riluzole and no deaths.^343^ But in a prospective, randomized controlled study of acute cervical SCI patients, administration of riluzole did not significantly improve neurological outcome/neuropathic pain.^344^

Minocycline targets multiple secondary injury mechanisms via its anti-inflammatory, antioxidant, and anti-apoptotic properties.^345^ Animal experiments showed that administration of minocycline result in preservation of the ultrastructure of spinal cord tissue,^346^ inhibition of microglial activation,^347^ significant improvement of motor function,^346^ reduce oligodendrocyte apoptosis and local inflammation,^348,349^ thus, improvement of motor function. But a contrary result was also reported.^350^ In a phase II placebo-controlled randomized trial of minocycline in acute SCI, no difference in motor recovery for thoracic SCI patients. In incomplete cervical SCI patient, functional outcomes exhibited differences but no statistical significance. No severe adverse event related to minocycline.^351^

#### Surgery

The aim of surgery for acute SCI is decompression and restoration of spinal alignment and stability.^352^ Studies have suggested that early decompression surgery may achieve reduced neural injury and improved outcomes, and early surgery may reduce the length of ICU stay and reduce post-injury medical complications.^353,354^ A multicenter, nonrandomized cohort study showed that early surgery achieves better neurological outcomes at the 6-month follow-up, as indicated by the odds of a 2-grade improvement in the AIS evaluation.^355^ Recently, a pooled analysis of individual patient data showed that surgical decompression within 24 h after acute SCI caused improved sensorimotor recovery. The first 24–36 h after acute injury is a crucial time window to achieve optimal neurological recovery with surgical decompression.^356^

In addition to external compression of the spinal cord, internal factors in the spinal cord itself can also affect clinical outcomes after surgery. Hematoma and edema after SCI can lead to increased intraspinal pressure, which may worsen the prognosis of SCI patients.^357,358^ After bony decompression, the intraspinal pressure remains high due to the tough and nondilated dura mater and has a tamponade effect on the blood vessels of the spinal cord, exacerbating blood supply issues and ultimately causing cytotoxicity and vasogenic edema.^62,358–360^ Therefore, surgical strategies to reduce intraspinal pressure have also been proposed. In vitro and in vivo animal experiments proved that durotomy can reduce intraspinal pressure and result in better blood perfusion, more neural tissue sparing and improved functional recovery.^361–364^ In clinical studies, Perkins and Deane performed durotomy in 6 neurologically impaired SCI patients with burst fracture after bony decompression, when the dura mater was noted to be tense and nonpulsatile. After the dura was incised, the return of dural pulsation was observed, and full or partial neurological recovery occurred in all the patients, as evaluated by Frankel grading. The authors believe that durotomy may be of some use in the recovery of spinal cord perfusion.^365^

Compared with durotomy, duroplasty can enlarge the dural space, reduce the intraspinal pressure and cause fewer complications, such as cerebrospinal fluid leakage, pseudomeningocele, and CNS infection, and it requires only 10–15 min of surgery time to suture an artificial dura to the dural margin.^357,358,360^ Phang et al conducted an open-label, prospective study comparing laminectomy+duroplasty versus laminectomy alone. The laminectomy+duroplasty group showed a greater increase in intradural space at the injury site, more effective decompression of the spinal cord, a lower intraspinal pressure and higher spinal cord perfusion pressure, and improved radiological and physiological parameters.^360^ In a retrospective analysis of 16 severe adult SCI cases without radiographic abnormalities, after durotomy with duraplasty, AIS scale and AISA scores improved, and the high level of intraspinal pressure after laminectomy continued to decrease steadily after surgery.^366^

Myelotomy or spinal cord incision was reported as early as 1911. Allen performed myelotomy in dogs with SCI, and function was restored. The author believed that myelotomy could drain the necrotic tissue.^367^ Hu et al. performed myelotomy on rats 24 h post-contusion SCI. Compared with those in the control group, significantly improved Basso, Beattie and Bresnahan scores, higher mean angle values in the incline plane test and edema were observed in the myelotomy group.^368^ Compared with durotomy only, durotomy plus myelotomy promoted spinal tissue formation, elicited a significant beneficial impact on gray matter sparing, increased the preservation of motor neurons and significantly promoted the recovery of hindlimb locomotor function.^363^ Four acute SCI patients received myelotomy within 24 h, and no patient developed new deficits postoperatively. All patients showed improvement in motor function of the upper extremities, and sensory disturbances also diminished to some degree.^369^ In another case report, a patient with central cervical SCI was treated by myelotomy. After recovering well from central cord syndrome, the patient developed rapidly progressive myelopathy 2 months after injury due to a new lesion at the C6 level rather than the original lesion at the C7 and T1 levels. Another myelotomy at the C6 level revealed intense gliosis inside the spinal cord. Rapid clinical improvement ensued. The authors concluded that secondary syringomyelia may be an end-stage condition after SCI and trigger a progressive, pathophysiological reaction, leading to central cord necrosis. They believe that in selected cases, myelotomy may interrupt this process.^370^ However, researchers also reported that compared to SCI-only animals receiving SCI, myelotomy 48 h after injury worsened Basso, Beattie, and Bresnahan score scores and did not improve plantar stepping, ladder climbing, urinary bladder voiding or sensory function, and no expected immunohistochemical changes were found.^371^ The negative effect after myelotomy may be due to the timing of surgery, as myelotomy itself can cause spinal cord damage, leading to aggravated inflammation in the injured spinal cord, which may cause negative outcomes.^357,358,371^

#### Rehabilitation treatment

##### Traditional rehabilitation

In hyperbaric oxygen (HBO) therapy, 100% oxygen is administered at a pressure onefold to threefold higher than atmospheric pressure. The underlying mechanisms for HBO include decreasing apoptosis and reducing inflammation and edema. Since ischemia is one of the major pathological changes after SCI, a high oxygen pressure, which increases oxygen tension in the spinal cord, may reduce the degree of ischemic injury in the spinal cord and improve clinical outcomes.^372^ Tan et al. published a retrospective study to assess the therapeutic effect of HBO therapy in the early treatment of acute SCI. Significantly improved ASIA scores and Frankel scores were found in the HBO group, and better results of MRI and electrophysiology tests were also reported.^373^ Another retrospective study of incomplete cervical SCI treated with and without HBO after surgery showed the safety and efficacy of HBO therapy and indicated that the longer the treatment lasts, the better the effects.^374^ Asamoto et al also reported that in the HBO group, the improvement rate indicated effectiveness in acute traumatic cervical SCI according to the Neurological Cervical Spine Scale (NCSS).^375^

Exercise is a noninvasive treatment that provides stimulation to certain regions of the spinal cord and appears to have multiple applications and benefits for SCI.^376^ Exercise has been proven to preserve muscle mass and strength, restore motor and sensory function, reduce local inflammation of the spinal cord, etc. After aerobic exercise, resistance training and combined exercises and in some studies of gait training and balance training, positive effects were observed.^377^ A home-based 6-week upper-body exercise improved indices of health-related quality of life in SCI patients, and the improvements were associated with increases in exercise self-efficacy.^378^

##### New means of rehabilitation

Neuromodulation technology, such as functional electrical stimulation (NCT03439319), can be used to improve limb function.^379^ The integrated field of medicine and industry has developed rapidly and innovated constantly, providing more advanced and convenient approaches for the rehabilitation of SCI patients, such as exoskeleton robots and brain-computer interfaces (BCIs). BCI is a new rehabilitation concept that bypasses the relay station of the spinal cord and directly allows an electroencephalogram to control the movement of limbs.^380–382^ In a primate SCI model, a brain-spine interface restored weight-bearing locomotion of the paralyzed leg on a treadmill and overground.^382^ A clinical trial on BCI has been carried out, and the results showed that hand motor function and tactility were recovered in patients with clinical complete SCI (NCT01997125).^380^ Another clinical trial showed that the neural activities of the motor cortex can be decoded into handwritten actions through BCI, significantly improving the writing speed and accuracy of paralyzed hands in patients with SCI (NCT00912041).^383^

#### Treatment outcomes and prognostic predictions

In terms of prognosis, it has been reported that the mortality of SCI is still high in recent years. SCI mortality rates in developed countries ranged from 3.1 to 22.2%, whereas mortality rates in developing countries ranged from 1.4 to 20.0%.^48,384–388^ Most patients can live a long time due to advancements in intervention and support techniques, but due to long-term bed rest or restricted activities, patients often experience unavoidable complications such as nonneuropathic pain, pendant pneumonia, bedsores and urinary tract infection.^389–393^ The prognosis of SCI is significantly associated with the site and severity of injury. After the basic vital signs have been stabilized, a thorough neurological evaluation is critical for the diagnosis and management of SCI patients. The Neurological and Functional Classification Standard of the American Spinal Injury Association (ASIA) is the preferred tool recommended by current guidelines and is an important tool for initial neurological examination and prognosis follow-up examination.^394,395^ There is another tool, named the SCI or Dysfunction Quality of Life Rating Scale (SCIDQLRS) (IANR 2022 version), which was designed as a single method to assess various items related to quality of life after SCI.^396^ It has been reported that 80% of ASIA grade A patients may not recover function and that 54% of ASIA grade B patients and 86% of ASIA grade C-D patients will have varied degrees of neurological recovery.^397,398^ A combination of assisted physiological tests, such as electrophysiological examination, can better predict the prognosis of SCI patients.^399–401^ Furthermore, age is also a predictive factor for SCI. It has been reported that 91% of patients with central SCI under 50 years of age regained walking ability, but only 41% of patients over 50 years of age regained walking capacity.^400,401^ As a result, making a thorough clinical decision based on a number of clinical laboratory test signs and long-term follow-up is required rather than merely predicting the prognosis of patients based on the ASIA scale.^395,402,403^ In addition, it should be noted that no studies have offered a molecular classification that can predict SCI risk and prognosis.

### Advanced technology in clinical trials

#### Clinical trial of cell transplantation in SCI

Cell transplantation to repair SCI is considered to be the most promising therapeutic strategy, and the cell types transplanted include MSCs, OECs, OPCs, NSCs/NPCs, ESCs, and iPSCs. Autologous stem cell transplantation has low risks of immunogenicity and tumorigenicity, while allogeneic cells are easy to obtain and expand, which is convenient for quality management. The safety, efficiency, cost, and feasibility of large-scale manufacturing should be considered. Several reports have compared different cell sources for SCI therapy.^404–406^ However, it is still unclear which one is the most effective for SCI therapy. MSCs can participate in immune regulation and neuroprotection to reduce cell loss. OECs/OPCs show advantages in myelin production and tissue modification. NSCs/NPCs may differentiate into neurons and replace the damaged cells to form a local network at the site of injury and form connections with intrinsic neurons. Various types of stem cells have been demonstrated to be safe and effective in rodents, dogs and nonhuman primates.^407–411^

ESCs and iPSCs are rarely directly used for transplantation because of their tumorigenicity, but their derivatives have been used in clinical trials of SCI. Human ESC-derived OPCs (LCTOPC1; previously known as GRNOPC1 and AST-OPC1) have been approved for clinical trials in the United States, and the results of the first clinical trial in 25 patients with subacute cervical SCI showed that 96% of the patients recovered one or more levels of neurological function and 32% recovered two or more levels.^412^ The transplantation of iPSC-derived NSCs/NPCs for subacute complete SCI was first approved in Japan (Table 3).^413^

**Table 3:** clinical trials about cell transplantation

Cell sources	Patients condition	Route of admin	Quantity and times	Subjects	Phase	ClinicalTrials.gov identifier or refs.
AST-OPC1	Subacute cervical SCI	Intraparenchymal	1 time; 2×10^6^/1×10^7^/2×10^7^	25	Phase 1/2a	NCT02302157^412^
hiPSC-NS/PC	Subacute SCI	Intralesional	1 time; 2×10^6^	4	Phase 1/2	jRCTa031190228.^413^
hNSPCs	Traumatic cervical SCI	Intralesional	1 time; 1×10^7^	19	Phase 1/2	KCT0000879^414^
human spinal cord-derived NPCs	Chronic SCI	Intralesional	1 time; 1.2×10^6^	4	Phase 1	NCT 01772810^416^
HuCNS-SC	Chronic cervical SCI	Perilesional intramedullary	1 time; 4×10^7^	52	Phase 2	NCT02163876^415^
Autologous BMSCs	Chronic and subacute SCI	Intrathecal	2–3 times; 1.2×10^6^/kg	9	Phase 1	NCT02482194.^418^
Allogeneic hUC-MSCs	Chronic SCI	Subarachnoid	4 times; 1×10^6^/kg	143	Phase 1/2	^419^
BMSCs	Chronic SCI (ASIA grad B)	Intramedullary+ intrathecal	1 time; 1.6×10^7^ (intramedullary)+ 3.2×10^7^ (intrathecal)	16	Phase 3	NCT01676441^421^
BMSCs+SCs	Complete SCI (3–12 m,ASI A)	Intrathecal	5×10^7^ BMSCs+5×10^7^ SCs	11	/	^422^
Autologous BMSCs	Chronic complete SCI	Intralesional	1 time; 5×10^6^/cm^3^	14	Phase 1	NCT01325103^508^
BMSCs	Chronic SCI	Intrathecal	1–8 times; 2×10^6^/kg each month	70	Phase 1/2	NCT00816803^509^
Autologous ADMCs	Chronic SCI	Intravenous	1 time; 4×10^8^	8	Phase 1	NCT01274975^510^
UCMSCs	SCI	Intralesional	1 time; 4×10^7^	34	Phase 3	NCT01393977^511^
Human UCMSCs	Chronic SCI	Intrathecal	4 times 1×10^6^/kg each month	66	Phase 2	NCT03521323^512^
UCMSCs	SCI	Intravenous+ intrathecal	1 time, 30 ml iv; 3times, 5×10^4^ intrathecal each week	7	/	^424^
BMMCs	SCI	Intrathecal	1 time; 2–4 ml cells	10	Phase 1/2	^426^
UCB-MNC	Chronic complete SCI	Perilesional intramedullary	1 time; 1.6×10^7^ ~ 6.4×10^7^	28	Phase 1/2	NCT01046786/ NCT01354483^427^

NSCs/NPCs for transplantation are mostly obtained from aborted fetuses. A phase I/IIa open-label and nonrandomized controlled clinical trial on the transplantation of human fetal brain-derived NSCs/NPCs into traumatic SCI patients showed that the AIS grade improved in 5 of 19 transplanted patients without serious adverse events.^414^ NSC transplantation was also reported in chronic SCI. Levi et al. carried out a phase II clinical trial (NCT02163876) using human NSCs (HuCNS-SCs), which were authorized as an investigational new drug (IND-15712) by the United States FDA, and observed recovery in the upper extremities.^415^ The authors also provided useful data on the surgical safety profile and feasibility of multiple intramedullary perilesional injections of HuCNS-SCs after SCI in another publication.^326^ Our team is conducting a clinical trial of intrathecal injection of aborted fetus-derived NSCs in the treatment of SCI (ChiCTR2200059595). Transplantation of human fetal spinal cord-derived NPCs (NSI-566) into SCI patients was also carried out, and the results showed that two of four subjects exhibited neurological improvement (Table 3).^416^

MSCs were used earlier and more widely in clinical trials of SCI due to their immunomodulatory mechanism, low immunogenicity, ease of acquisition, and fewer ethical restrictions.^417,418^ A phase 1/2 pilot study of 143 SCI patients showed that repeated subarachnoid administration of allogeneic human UCMSCs significantly improved pinprick, light touch, motor and sphincter scores.^419^ However, there are still questions and limitations in this field.^420,421^ Combined transplantation of different types of cells, transplantation of MSCs combined with biomaterials, and comprehensive rehabilitation may be strategies to improve the effect of MSCs in clinical trials.^422–425^ A phase 1/2 clinical trial of cell transplantation combining human autologous Schwann cells and BMSCs was carried out in subacute complete SCI patients and revealed statistically significant improvements in sensory and neurological functions (Table 3).^422^

Bone marrow mononuclear cells (BMMCs) and umbilical cord blood-derived mononuclear cells (UCB-MNCs) are both useful cell types for repairing SCI, and their function has been verified in clinical research on SCI (Table 3).^426,427^

#### Clinical trial of biomaterials in SCI

Biomaterials have been used to replace PNS grafts, which can achieve significant functional recovery by improving axonal regeneration when NPCs are seeded.^428^ To overcome the solid morphology of scaffolds, hydrogels and self-assembling peptides have been developed to provide injectable scaffolds.^429–432^ These biomaterials can be modified to deliver drugs or stem cells and have been shown to provide functional recovery in rodent models of SCI.^23,433–437^

However, there are few clinical studies on functional material transplantation to repair SCI. Dai et al. transplanted NeuroRegen scaffolds combined with UCMSCs into two acute complete SCI patients, and the results showed significant recovery in sensory and motor functions and improvement in bowel and bladder function (NCT02510365).^423^ Another 3-year clinical study performed by Dai’s team enrolled seven acute complete SCI patients, and neuro-Regen scaffolds loaded with autologous bone marrow mononuclear cells (BMMCs) were implanted into the site after the necrotic spinal cord tissue was surgically cleaned under intraoperative neurophysiological monitoring. No adverse symptoms were observed, and partial shallow sensory and autonomic nervous functional improvements were observed in some patients, but no motor function recovery was observed (NCT02510365).^438^ These findings indicate that implantation of biomaterials combined with stem cells may serve as a safe and promising clinical treatment for patients with acute complete SCI. However, based on the current development trends, multidimensional therapy based on biomaterials, stem cells, cytokines, physical factors, and rehabilitation has great potential in the regeneration and repair of SCI. To date, some scaffolds based on nanotechnology have entered the clinical trial stage and have yielded some evidence of safety and efficacy, but a large amount of supporting data is still needed before large-scale clinical translation.

#### Clinical trial of physical regulation in SCI

##### Ultrasound

Recent clinical trials of ultrasound have mostly focused on the evaluation and complications therapy for SCI. Trials were designed to establish whether the change in ultrasound muscle parameters from the baseline correlates with functional status of SCI patients (in comparison to rehabilitation) (NCT04303728) or to assess blood flow in the injured area of the spinal cord (NCT04056988) or predict deep vein thrombosis in SCI patients (NCT02796235). Ultrasound has also been used to assess neurogenic bladder function after SCI (NCT01299792, NCT01297673).

In addition, ultrasound is helpful for treating complications of SCI. A pilot study evaluated ultrasound/ultraviolet-C and laser use for the treatment of pressure ulcers in patients with SCI and found that ultrasound/ultraviolet-C may decrease healing time and allow faster return to rehabilitation programs, work, and leisure activities among patients with SCI who have pressure ulcers.^439^ Another trial investigated the effect of one-time shock wave therapy (ESWT) on lower limb spasticity in patients with incomplete SCI (NCT02203994). Furthermore, ultrasound can be used as guidance for corticosteroid injection or microfragmented adipose tissue injection.^440,441^ In clinical trials, there has been few research on neural regeneration and neural circuit remodeling after ultrasonic stimulation in SCI. One clinical trial involving 82 patients with SCI found that extracorporeal shock waves can change the cell response and reduce neuron loss (NCT04474106).^442^

##### Magnetic field control

Most clinical trials in this area have been associated with transcranial magnetic stimulation (NCT02914418, NCT02914418, and NCT04372134). In the context of incomplete SCI, 15 daily sessions of high-frequency rTMS can improve motor scores, walking speed, and spasticity in the lower limbs.^443^ Another double-blind, randomized sham-controlled crossover trial showed that rTMS produced positive results in treating individuals with physical impairments.^444^ In addition, recent findings indicate that 10 Hz rTMS over the hand area of the motor cortex could alleviate acute central neuropathic pain during the early phase of SCI and could enhance MEP parameters and modulate BDNF and NGF secretion. The analgesia-enhancing effects of high-frequency rTMS might be related to the amelioration of M1 and PMC hypersensitivity, shedding light upon the clinical treatment of SCI-related neuropathic pain.^445,446^

Other studies have focused on magnetic stimulation for complications post SCI. A randomized controlled trial showed the effects of repetitive transcranial magnetic stimulation on recovery of lower limb muscle strength and gait function following SCI.^447^ For bladder dysfunction after SCI, a clinical trial investigated the changes in bladder function in response to long-term bladder conditioning by FMS to further optimize FMS technology and parameters for effective bladder emptying in SCI (NCT00011557). Another trial focused on repetitive transcranial magnetic stimulation and pelvic floor muscle training for female neurogenic bladder dysfunction after SCI (ChiCTR1900026126).^448^

Neuropathic pain after SCI is also a concern (NCT01932905). Studies have attempted to use a combination of high-frequency noninvasive rTMS and exercise training to enhance motor recovery (NCT01915095) and to investigate the effects of repetitive transcranial magnetic stimulation combined with transspinal electrical stimulation (tsES) intervention on cortical excitability, brain structure, and lower extremity motor ability in individuals with incomplete SCI (NCT04194099).

A brain-computer interface-based medical device based on electromagnetic field (EMF) stimulation was recently invented, and this device is being used to investigate the safety and efficacy of the new advanced electromagnetic field therapy in the management of chronic SCI patients (NCT04050696).

##### Electric control

Functional electrical stimulation technology (FES), transcutaneous electrical nerve stimulation (TENS) and epidural electrical stimulation (EES) are the most widely used electrical stimulation technologies in clinical trials.

FES is a surface electrical stimulation technology that generates a series of electrical stimuli that trigger action potentials in intact peripheral nerves to further activate muscle contractions.^449^ FES has advantages in improving muscle status after SCI, but there is no clear report on neural regeneration and nerve remodeling after SCI.^450^ Advanced weight-bearing mat exercises combined with functional electrical stimulation were shown to improve the ability of wheelchair-dependent people with SCI to transfer and achieve independence in activities of daily living.^451^ The combination of FES with resistance training may enhance oxygen uptake and ventilatory efficiency independent of mitochondrial complexes after SCI.^452^

TENS is another noninvasive therapeutic modality that is commonly used in pain control and exerts its effects by stimulating large-diameter mechanosensitive afferent nerve fibers in the skin.^453^ A clinical study compared the effects of TENS and FES on lower limb spasticity in patients with SCI, and the results suggest that both TENS and FES have the potential to be used as adjunct therapies to relieve spasticity in the clinic, and FES may have better effects on patients presenting with spastic reflexes.^450^ Another clinical study reported that TENS enabled 8 children with trunk control disorder due to acquired SCI to sit upright (NCT03975634).^454^ TENS combined with training improves hand strength and manual dexterity in subjects with SCI.^455^ The lumbosacral spinal networks can be modulated transcutaneously using electrical spinal stimulation to facilitate self-assisted standing after chronic motor and sensory complete paralysis.^456^ A new pilot clinical trial has been started to explore the efficacy of transcutaneous spinal cord stimulation (TESCoN, SpineX Inc., CA, USA) in mitigating crucial autonomic dysfunctions that impact the health-related quality of life of individuals with SCI (NCT05369520).

Compared with FES and TENS, EES is an invasive electrical stimulation technique. An electrical stimulation device is surgically placed on the epidural area corresponding to the target area. In 2011, Harkema’s team reported in the Lancet that EES promoted standing in patients with SCI with complete motor impairment.^457^ Many studies have confirmed that EES promotes functional recovery after SCI and improves the quality of life, including motor functions and hemodynamics.^458–460^ In one report, four patients with chronic motor complete SCI achieved independent standing and trunk stability via EES combined with rehabilitation.^238^ In another report, three patients with severe chronic cervical SCI achieved voluntary control of walking with an implanted pulse generator through targeted spinal cord stimulation. All these studies suggested that EES is a very promising method for the treatment of SCI. Deep brain stimulation (DBS) is another invasive technology for locomotion recovery. A clinical trial was carried out on patients with a DBS implant at the site of SCI, and an electrode to stimulate the midbrain motor area was designed (NCT03053791).^461^ Due to the rapid development of computer technology, electrocorticography (ECoG)-based brain-computer interface systems can measure brain activity using electrodes implanted on the surface of the brain. Preliminary results demonstrate the feasibility of ECoG-based systems for individuals with paralysis (NCT01393444).^462^ The above information is shown in the table below (Table 4).

**Table 4:** clinical trials about physical regulation in SCI

Physical factors	Patients condition	Stimulus method	Stimulation parameters and frequency	Subjects	Phase	ClinicalTrials.gov identifier or refs
Ultrasound and ultraviolet-C (US/UVC)	Pressure ulcers post SCI	External	US: 3 MHz, 0.2 W/cm^2^ (1:4 pulse ratio) for 5 min UVC: (95% emission at 250 nm) calculated level	20	N/A	^439^
Ultrasound	SCI (AIS C and D)	External	energy level: 0.030 mJ/mm^2^, frequency: 4 Hz 2000 pulses per muscle	20	N/A	NCT02203994.
Ultrasound	Traumatic spinal injuries	External	Energy level: 0.1–0.19 mJ/mm^2^, frequency: 2–5 Hz	82	N/A	NCT04474106^442^
rTMS	Incomplete SCI	External	5 Hz, 12 trains of 50 magnetic pulses	20	N/A	NCT02899637^444^
rTMS	Complete and incomplete SCI	External	10 Hz, 1500 pulses	48	N/A	^445^
rTMS	Neuropathic pain following SCI	External	10 Hz, 1200 pulses	21	N/A	^446^
rTMS	chronic SCI	External	20 Hz, 1800 pulses	20	N/A	^447^
FMS	SCI above T10 level, six months post injury	External	N/A	36	Phase 2	NCT00011557
rTMS	Chronic SCI	External	5 Hz for 20 min	44	N/A	ChiCTR1900026126^448^
rTMS	6 months after SCI	External	5 Hz, 10 trains of 15 pulses	42	N/A	NCT01915095
rTMS and tsES	Incomplete SCI (AIS B to D)	External	20 Hz rTMS, 1200 s (Brain) 2.5-mA tsDCS, 600 s (Spinal)	12	N/A	NCT04194099
Electromagnetic Field (EMF)	Incomplete SCI (AIS B to D)	External	BQ, a brain-computer interface device (BCI)	8	N/A	NCT04050696
FES	Chronic SCI	External	pulse width 400 µs, frequency 40 Hz, on/off cycle 5/10 s	16	N/A	^451^
FES	Traumatic SCI	External	pulse width 450 µs, frequency 30 Hz	23	N/A	^452^
TENS	Chronic SCI	External	pulse width 1 ms, frequency 15–30 Hz. (T11 at 30 Hz, L1 at 15 Hz, and C5 at 30 Hz)	8	N/A	NCT03975634^454^
TENS	Complete cervical SCI	External	10 kHz with a 16-channel A/D board	12	Phase I/II	NCT02313194^455^
Transcutaneous spinal cord stimulation (TCSCS)	Chronic traumatic SCI	External	frequency between 1 Hz and 90 Hz amplitude 1.0–3.5 mm	30	N/A	NCT05369520
EES	Traumatic SCI	Implanted	2 Hz, 450 μs with 0.1 V intervals ramping incrementally	4	N/A	NCT02339233^238^
EES	Chronic cervical SCI	Implanted	Programmed, 20–100 Hz, 1.8–2.7 mA	3	N/A	^458^
DBS	Incomplete SCI AIS C	Implanted	20 Hz and 400 µs pulse	5	Phase I/II	NCT03053791^461^
Electrocorticography (ECoG)	Complete cervical SCI	Implanted	Brain-computer interface (BCI) system, 0–200 Hz frequency range (25th order, 10 Hz frequency bands) using 300 ms	3	N/A	NCT01393444^462^

## Perspectives

With the deepening of the understanding of molecular pathological mechanisms after SCI, different types of intervention strategies, such as controlling inflammation, reducing cell death, modulating scar formation, and regulating neurotrophic factors and angiogenesis, have been validated in animal models in terms of safety and efficacy. However, SCI is still a tough challenge, mainly due to the following reasons. First, various pathological mechanisms of SCI have temporal and spatial characteristics, and they are interlinked and interact with each other, which are difficult to clearly describe and elaborate. Second, the limitation of neural regeneration ability still lacks effective improvement methods; the bottleneck of effective nerve regeneration is still difficult to break. Third, due to the difference of species, it is difficult to translate the research results obtained in animal models into clinical applications.

Although many strategies show reassuring results in animal models of SCI, however, these experiments involved very controlled injuries and recovery conditions in animals while hardly matched for age, weight, gender, species, and genetic background. This obviously dwarfed in comparison to the natural variability that occurs in the acute human SCI. In addition, the stability of animal models is still facing huge challenges. Different modeling methods can only imitate the injury mechanism of SCI to a certain extent, but cannot fully reflect the real molecular pathological timing characteristics of SCI. There are few relevant detection methods for human SCI at present. Blood and cerebrospinal fluid are routine detection methods. In the future, there is an urgent need to develop technical means that can noninvasive or minimally invasive detect the true molecular pathological process of patients with SCI.

The appreciation of the heterogeneity of human SCI is partly the result of the challenges that have been experienced in the execution of clinical trials of novel therapies, particularly in the acute situation. As variability in patients with SCI, the sample size should be increased as much as possible in clinical research to reduce the research bias. Almost all acute SCI studies are faced with the difficulty of recruiting patients. The failure to recruit enough patients within the specified time has led to the suspension or cancellation of many clinical studies on acute SCI. In the future, more network-based technologies need to be developed to overcome this problem to promote clinical research of SCI.

Moreover, it is necessary to further optimize the clinical research of SCI. Due to the high heterogeneity of SCI and the large individual differences, the inclusion window can be appropriately reduced, and the objective biological indicators of the severity of the injury and the more accurate prognosis analysis system of SCI can be used to recruit a precisely defined population.

In the future, the pathological mechanism still needs more in-depth research. Single-cell sequencing technology has already revealed many cell subsets or subtypes, which may become key intervention targets, but there is still a lack of confirmatory research results.^33,58,134,463^ In addition, quantitative pharmacology, spinal cord organoids, the intestinal flora-mediated “brain gut axis” and quantum mechanics-mediated neurobiology are new perspectives for studying the pathological mechanism of SCI.^464^

Although stem cell transplantation for SCI is an important therapeutic strategy, many challenges remain. The poor effect and unclear time window of stem cell transplantation, the associated tumorigenic risk, the immune response after transplantation, and the cell survival rate in the human body are issues that need to be carefully considered. Although the safety and efficacy of stem cells have been demonstrated in animal experiments, heterogeneous results remind us that more preclinical studies are needed to evaluate the safety and efficacy of stem cell therapy.^407^ Future stem cell therapy for patients with SCI should be based on the pathological characteristics of SCI and the cellular and molecular characteristics of the stem cells themselves and should meet the requirements of easy clinical transformation, that is, the ability to promote the reconstruction of neural circuits or the ability to repair the microenvironment.^465,466^ It is worth mentioning that, from the perspective of neurodevelopment, it is possible to screen the cell types most suitable for the regeneration and repair of SCI.^467,468^ More importantly, research on the clinical transformation of stem cells requires social, policy and legal support.

The spinal cord tissue is surrounded by cerebrospinal fluid and protected by bony structures in the spinal canal; it is soft in nature and passes up and down to mediate signal connections between the brain and different segments of the spinal cord.^469–471^ Therefore, considering the current understanding of the characteristics of the spinal cord, and in the future, more excellent biological scaffold need to be developed and should meet the following conditions: (1) good biocompatibility to promote neuron adhesion; (2) a high water content to meet the requirements of cell metabolism; (3) a three-dimensional fiber structure with high permeability and an appropriate orientation for cell migration and axon growth; and (4) excellent flexibility to resist deformation under various stresses. Thus, the mechanical properties and microstructure of such a scaffold still need to meet the requirements for the active repair and regeneration of SCI. To achieve better treatment of SCI with biomaterials, the active combination of cell therapy and biomaterials via tissue engineering is urgently needed, thus providing alternative technologies such as 3D bioprinting and microfluidic devices.^472–477^

It is difficult to effectively promote recovery after SCI by relying on a single intervention strategy, and future clinical intervention approaches may include multiple synergistic intervention strategies. It is necessary to promote cooperation among all fields of neuroscience to form a comprehensive treatment strategy that integrates surgical and biological rehabilitation.^478,479^ In addition, attention should be given to psychological interventions for patients with SCI because of its high disability rate. Rehabilitation and regeneration are equally important for patients with SCI. In the future, artificial intelligence will be combined with rehabilitation to make more breakthroughs, such as brain-computer interfaces, intelligent devices, and quantum technology.^480^
